# Nitric oxide charged catheters as a potential strategy for prevention of hospital acquired infections

**DOI:** 10.1371/journal.pone.0174443

**Published:** 2017-04-14

**Authors:** David Margel, Mark Mizrahi, Gili Regev-Shoshani, Mary KO, Maya Moshe, Rachel Ozalvo, Liat Shavit-Grievink, Jack Baniel, Daniel Kedar, Ofer Yossepowitch, David Lifshitz, Andrei Nadu, David Greenberg, Yossef Av-Gay

**Affiliations:** 1 Division of Urology, Rabin Medical Center and Tel-Aviv University, Sackler School of Medicine, Petach-Tikva, Israel; 2 ENOX Ltd., Habarzel 38, Tel-Aviv, Israel; 3 Department of Medicine, Division of Infectious Disease, University of British Columbia, British Columbia Province, Vancouver, Canada; Azienda Ospedaliera Universitaria di Perugia, ITALY

## Abstract

**Background:**

Catheter-Associated Hospital-Acquired Infections (HAI's) are caused by biofilm-forming bacteria. Using a novel approach, we generated anti-infective barrier on catheters by charging them with Nitric Oxide (NO), a naturally-produced gas molecule. NO is slowly released from the catheter upon contact with physiological fluids, and prevents bacterial colonization and biofilm formation onto catheter surfaces.

**Aims and methods:**

The aim of the study was to assess the anti-infective properties of NO-charged catheters exposed to low concentration (up to 10^3^ CFU/ml) of microbial cells *in-vitro*. We assessed NO-charged tracheal tubes using *Pseudomonas aeruginosa*, dialysis and biliary catheters using *Escherichia coli*, and urinary catheters using *E*. *coli*, *Candida albicans* or *Enterococcus faecalis*. Safety and tolerability of NO-charged urinary catheters were evaluated in a phase 1 clinical study in 12 patients. Six patients were catheterized with NO-charged catheters (NO-group), followed by 6 patients catheterized with regular control catheters (CT-group). Comparison of safety parameters between the study groups was performed.

**Results:**

NO-charged tracheal, dialysis biliary and urinary catheters prevented *P*. *aerugino*sa, *E*. *coli* and *C*. *albicans* attachment and colonization onto their surfaces and eradicated corresponding planktonic microbial cells in the surrounding media after 24–48 hours, while *E*. *faecalis* colonization onto urinary catheters was reduced by 1 log compared to controls. All patients catheterized with an NO-charged urinary catheter successfully completed the study without experiencing NO-related AE's or serious AE's (SAE's).

**Conclusion:**

These data highlight the potential of NO-based technology as potential platform for preventing catheter-associated HAI's.

## Introduction

Hospital-Acquired Infections (HAI's) affects millions of patients worldwide annually, resulting in increased morbidity and mortality, and inflicting substantial financial losses to health systems [1]. High frequency of HAI's such as Blood Stream Infection (BSI), Urinary Tract Infection (UTI) and Ventilator-Associated Pneumonia (VAP) are associated with the use of invasive medical devices such as central lines, urinary catheters and tracheal catheters, respectively [2, 3].

Bacteria can adhere to the surface of the catheter within hours from catheterization and colonize, creating a persistent environment called a biofilm [4–6]. These colonies consist of bacteria that have a profoundly different set of genes from those of planktonic bacteria [6], and resistant to standard antibiotics, disinfectants or germicides [7–8], augmenting the potential of these pathogens to cause infections in catheterized patients.

Nitric Oxide (NO) is a naturally-produced gas molecule which plays an important role in host-defense against a large variety of pathogens [9]. Several NO-releasing technologies were developed during the years. Amongst them NO-releasing nanoparticles and dendrimers were shown to have anti-microbial and anti-biofilm capabilities against multiple microbial species [10–12].

Using a novel approach, we generated an anti-infective barrier on catheters by charging them with NO. We have previously shown *in-vitro* that NO-charged urinary catheters that are immersed in urine slowly release low concentration of NO (μM levels) for at least 14 days, and prevent *Escherichia coli* colonization and biofilm formation onto their luminal and exterior surfaces [13]. NO-charged urinary catheters were found effective also in comparison with antibiotic-coated catheters (Nitrofurazone) and eradicated all bacteria in planktonic and biofilm states [14]. Moreover, NO-charged urinary catheters were superior compared to silver-coated catheters (that are clinically used) in preventing bacterial colonization and biofilm formation onto catheters surfaces [14].

Although NO is a natural gas and has a short half-life, prior studies demonstrated it may cause several unwanted side effects. NO gas at low concentrations of 5–40 ppm was found to be associated with decrease in blood pressure, inhibition of platelet aggregation, increased bleeding time and local side effects to its surrounding tissue such as uncontrolled erection (i.e. priapism) local skin irritation, and the formation of local skin edema and erythema [15–20].

The objectives of this study were to evaluate the anti-infective properties of several NO-charged catheter types *in-vitro* against low concentration (up to 10^3^ CFU/ml) of common gram-negative, gram-positive bacteria and fungi, and to evaluate for the first time in men the safety and tolerability of NO-charged urinary catheters in patients undergoing radical prostatectomy that were catheterized for a period of 7–28 days.

## Materials and methods

### Charging of catheters with NO

Four different types of catheters were charged with NO in a previously described exposure chamber under proprietary conditions and using a proprietary technique [21]. These catheters were: Mallinckrodt™ hi-contour tracheal tube 4,5 Oral/Nasal 6,2 (COVIDIEN), HemoStream™ chronic dialysis catheter 15.5Fr (Angiotech), SKATER™ biliary drainage catheter 12Fr (Angiotech) and 18Fr silicone urinary catheters (Biometrix, Ltd, Israel). All catheter types were used *in-vitro* and the urinary catheter was used *in-vivo*. For each catheter type we used uncharged catheters or catheter sections as controls.

### Measurement of NO release

To measure NO release following NO-charging each tracheal catheter was cut into 1-cm sections, and each dialysis, biliary and urinary catheter was cut into 2-cm sections. Each of the NO-charged and control catheter sections was placed in a tube and was immersed in sterile water. Tracheal catheter sections in 2.5ml, dialysis and biliary catheter sections in 4ml and urinary catheter sections in 2.25ml. Nitrite concentration in water was measured over 24 hours following immersion using Griess reagent [22]. Nitrite concentration (μM) was converted to NO concentration (ppm) as described elsewhere [13].

### Bacterial preparation

*Pseudomonas aeruginosa*, *E*. *coli*, *Enterococcus faecalis* and *Candida albicans* were obtained from American Type Culture Collection (ATCC #14210, #25922, #29212 and #90028 respectively). *P*. *aeruginosa* were grown in Muller Hinton Broth (MHB) to a concentration of 1×10^8^ CFU/ml and diluted with MHB to a working concentration of 10^2^ CFU/ml. *E*. *coli* were grown in Luria Broth (LB) to a concentration of 2.5×10^8^ CFU/ml and diluted with LB to working concentrations of 10^2^ and 10^3^ CFU/ml. *E*. *faecalis* and *C*. *albicans* were plated onto blood agar and sabouraud agar plates and incubated at 37°C for 24 and 48 hours, respectively. After incubation, *E*. *faecalis* and *C*. *albicans* colonies were taken and diluted with saline to a concentration of 1×10^8^ or 1×10^5^ (McFarland 0.5), respectively. Samples were further diluted with saline (2% LB) to a working concentration of 10^2^ CFU/ml.

### Microbial attachment and colonization onto catheters surfaces

2-cm sections of NO-charged and control catheters were immersed in suspensions contaminated with microbial cells; tracheal catheter sections were immersed in 1ml of MHB containing 10^2^ CFU/ml of *P*. *aeruginosa* and incubated for 48-hours at 30°C. Dialysis and biliary catheter sections were immersed in 1.5ml of LB, containing 10^2^, or 10^3^ CFU/ml of *E*. *coli* and incubated for 24-hour at 37°C. Urinary catheter sections were immersed in 2.25ml of saline (2% LB) containing 10^2^ CFU/ml of *E*.*coli*, *C*. *albicans* or *E*. *faecalis* and incubated for 24 hours at 37°C. Following incubation, each of the catheter sections was washed twice with sterile saline (0.9%). Then each of the catheter sections was qualitatively assessed for microbial attachment following rolling of the section onto Muller Hinton (MH) agar plates, LB agar plates, MacConkey agar plates or blood agar plates. Quantitative assessment for the number of colonizing microbial cells onto 1-cm of urinary catheter sections was performed after 24 hours of incubation with contaminated suspensions where sections were transferred into new tubes containing fresh saline solution and sonicated as described elsewhere [13].

### Planktonic anti-microbial activity of NO-charged catheters

2-cm sections of NO-charged and control tracheal and urinary catheters were immersed in 1ml of MHB containing 10^2^ CFU/ml of *P*. *aeruginosa* or 2.25ml of saline (2% LB) containing 10^2^ CFU/ml of *C*. *albicans* or *E*. *faecalis*, respectively. Tracheal catheters were incubated for 48 hours at 30°C, and urinary catheters were incubated for 24 hours at 37°C. After incubation, samples were vortexed and aliquots were plated onto MH agar plates, MacConkey agar plates or blood agar plates and incubated overnight at 37^0^ C. CFU's were counted and final bacterial load was calculated as CFU/ml.

### Clinical study population

Patients undergoing radical prostatectomy surgery at the Urological Department, Rabin Medical Center (RMC) were selected by the principal investigator and screened for their eligibility. **Inclusion criteria:** 1) Male patients (>18yr). 2) Life expectancy of more than 12 months. 3) Patients scheduled for radical prostatectomy surgery. 4) The patient is willing and able to read, understand and sign the study specific informed consent form. **Exclusion criteria:** 1) any underlying disease involving the heart, lungs, skin, immunodeficiency or infection that could influence study results. 2) A urinary culture demonstrating UTI before radical prostatectomy surgery. 3) Known urethral stricture and a history of recurrent UTI's. Patients who met all inclusion criteria and none of the exclusion criteria were invited to participate in the study.

### Ethics

This clinical study was conducted according to the laws, regulations and administrative provisions relating to the implementation of Good Clinical Practice (GCP) in the conduct of clinical trials on medicinal products for human use, as applicable by national legislation and according to the Israeli Ministry of Health (MOH) regulations. The clinical study was approved by the Investigational Review Board (IRB) of RMC and by the Israeli MOH. All patients eligible for participation in this study provided a written informed consent prior to enrollment.

### Clinical study design

**Fig 1:**
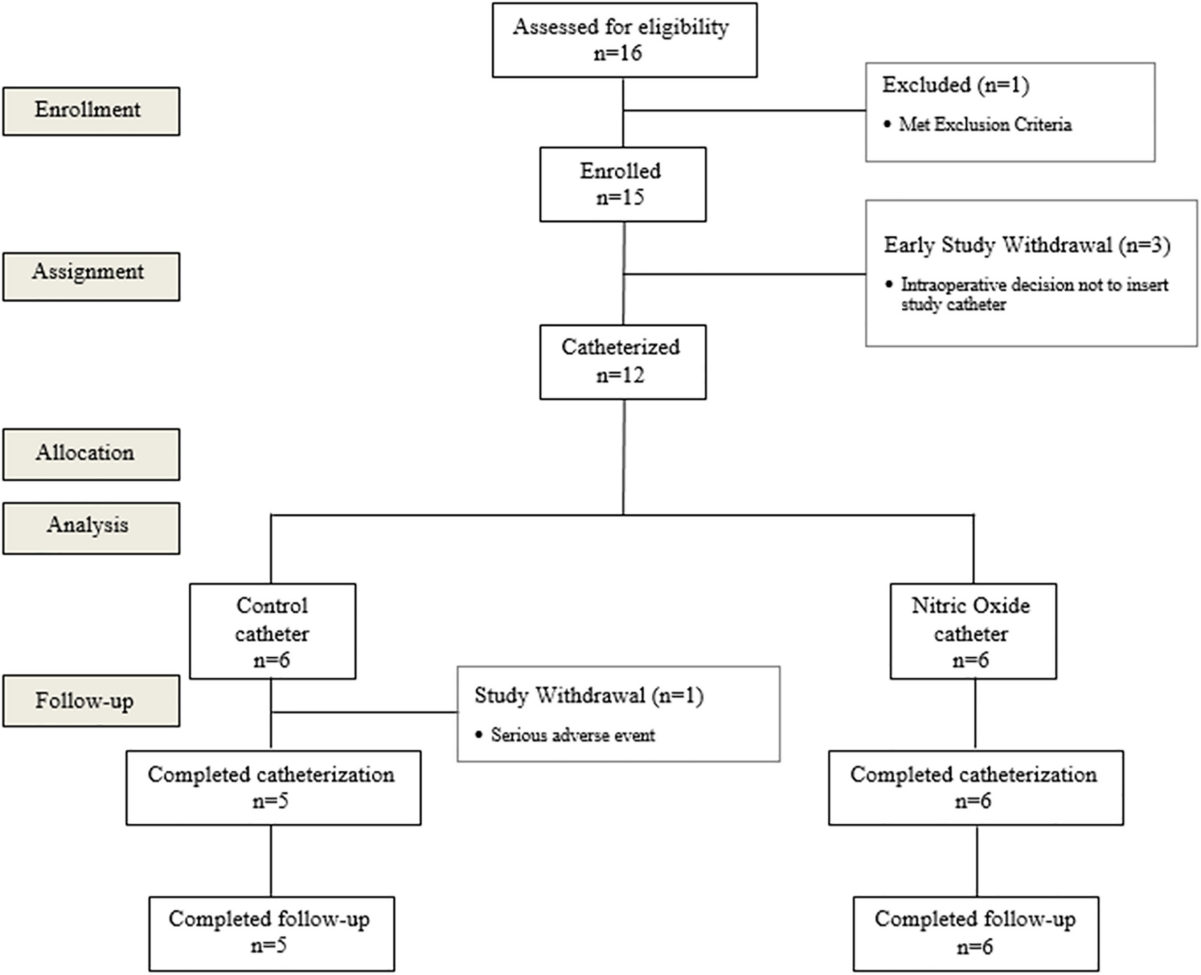
Patient disposition. A CONSORT flow diagram showing the number of patients assessed for eligibility, enrolled, assigned for catheterization, allocated to NO or CT group, analyzed and completed catheterization and follow-up. Patients excluded or withdrawn during the study are presented in dashed squares.

A prospective, phase I, open-label, case controlled single-center study performed at the Urology Department, Beilinson Hospital, RMC, Israel (registry name at ClinicalTrials.gov- NCT02277171) (Fig 1). Out of 15 patients enrolled (the number of enrolled patients was extended from 12 to 15 patients during the study, since 3 patients enrolled to the study were withdrawn prior to catheterization during radical prostatectomy surgery), 12 patients were catheterized with an 18Fr diameter silicone foley catheter for at least 1 day and were included in the study in the Intention To Treat (ITT) cohort. Six patients were catheterized with an NO-charged catheter (NO-group) during radical prostatectomy surgery. After all 6 patients from the NO-group completed the study, another group of 6 patients were catheterized during radical prostatectomy surgery with a regular non-charged control catheter (CT-group). Each patient was catheterized for 7–28 days, and was followed for 30–45 days after catheterization (catheterization time was extended during the study from 14 days to up to 28 days, and follow-up period was extended from 30 to up to 45 days after catheterization at the request of the urological department physicians). All catheterizations were performed by a physician. Patients from both groups received standard treatment in the hospitals urology department and in the outpatient clinic including anti-coagulation treatment prior to surgery and antibiotics during hospitalization. All deviations from protocol were approved by the IRB of RMC. Protocol was amended accordingly during the study and approved by IRB of RMC.

### Safety assessments

Number and proportion of possible AE's or SAE's that may be associated with NO-charged urinary catheters including decreased in blood pressure, inhibition of platelet aggregation, increase in bleeding time, and local side effects such as unwanted erection (i.e. priapism) and local skin irritation were assessed in each patient from the NO-group throughout the study and were compared to the CT-group.

During the study vital signs (i.e. Blood pressure, Temperature) and coagulation (i.e. Prothrombin Time–PT, activated Partial Thromboplastin Time–aPTT, International Normalized Ratio–INR), Hematology (i.e. Platelets, White Blood Cells–WBC, hemoglobin and Neutrophils) and biochemistry (i.e. C-reactive) tests that may be in association with NO-releasing urinary catheter were taken prior to catheterization at screening for baseline evaluation, at hospitalization (daily), at catheter removal day and at follow-up visit. A comparison of parameters values between the groups and changes in parameters values from baseline levels during the study within each study group were evaluated and compared at the 1^st^ day of catheterization (i.e. 24 hours after catheter insertion), at hospital discharge day, at catheter removal day and at follow-up visit. All lab personal were blinded to the study groups. All data for each individual patient was collected in paper Case Report Form (CRF). One copy of each CRF was kept by the investigator and the 2 copies were retrieved by the sponsor.

### Statistical analysis

Fisher's exact test was used for analyzing the difference in proportions between the NO-group and CT-group. The Paired T-test for two means was applied for analyzing changes in parameters within a study group. The Non-parametric Mann-Whitney U-test was applied for analyzing differences in parameters between the NO-group and CT-group.

All data analysis was conducted on the ITT cohort. Last Observation Carried Forward (LOCF) approach was applied for all patients catheterized for at least 1 day (ITT cohort) to account for missing data at or prior to study termination.

Although statistical tests were conducted to compare between the groups and to analyze changes from baseline measurements at different time points during the study, the planned sample size was not expected to show statistical significance or statistical power, only demonstrate a safety profile. The data was analyzed using GraphPad Prism® version 5.0.

## Results

### Release of NO from charged catheters

**Fig 2:**
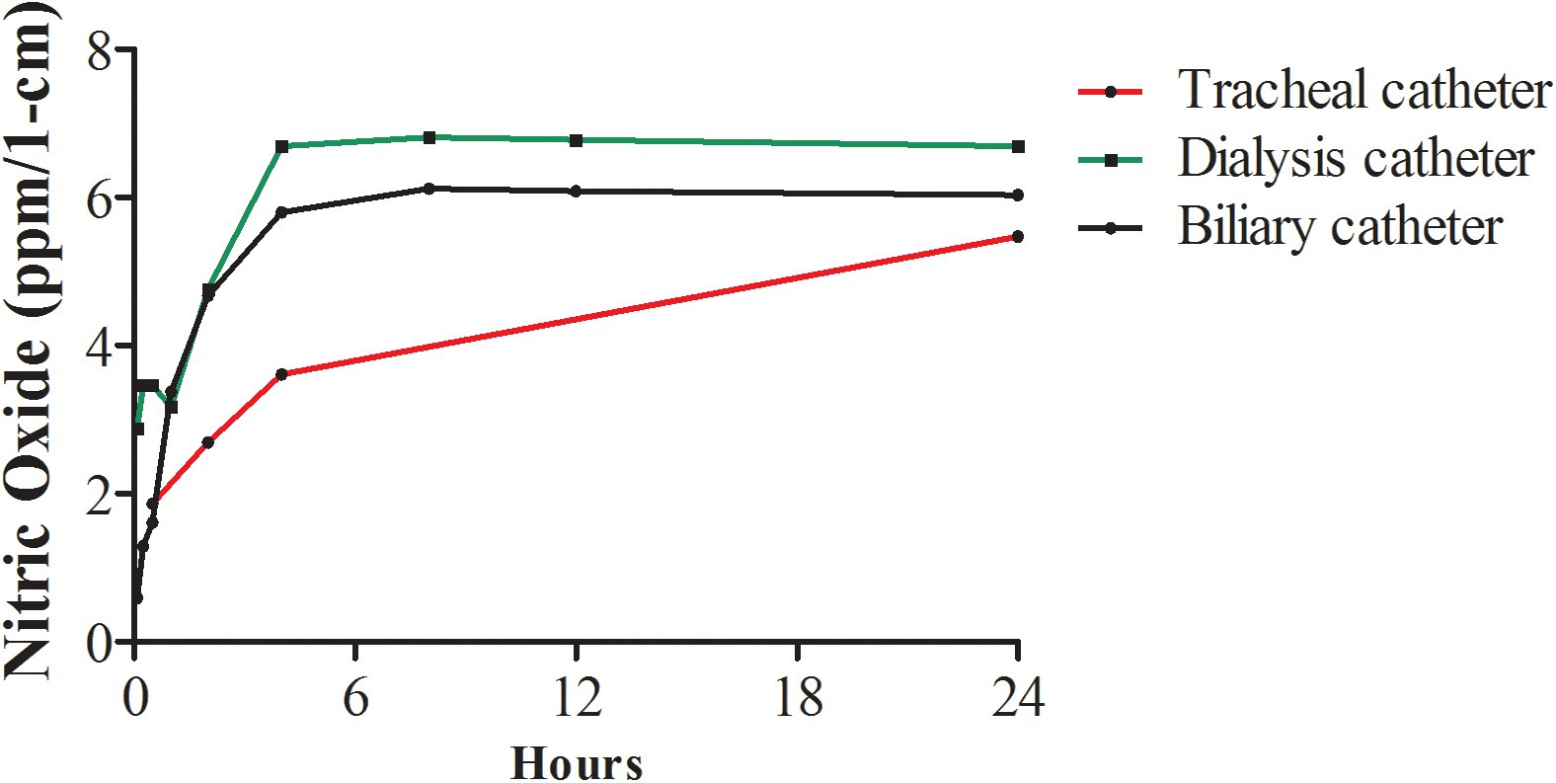
Release of NO from charged catheters. Twenty four hour accumulation of NO in water after release from charged tracheal, dialysis, biliary and urinary catheters (*n* = 3 for each catheter). NO levels were determined using Griess reaction.

NO has a short half-life *in-vivo* of a few seconds. Therefore, the level of more stable NO metabolite, nitrites, was used for indirect measurement of NO in fluids. As shown (Fig 2) NO release from tracheal, dialysis and biliary catheters was measured during 24 hours, with the largest amounts released during the first hours of immersion in water. After 24 hours the NO levels released per 1-cm of each of the NO-charged tracheal, dialysis and biliary catheters were 5.47 ppm, 6.69 ppm and 6.02 ppm, respectively. NO was not released from non-charged control sections (not shown).

### NO-charged catheters prevent/reduce microbial attachment and colonization

**Fig 3:**
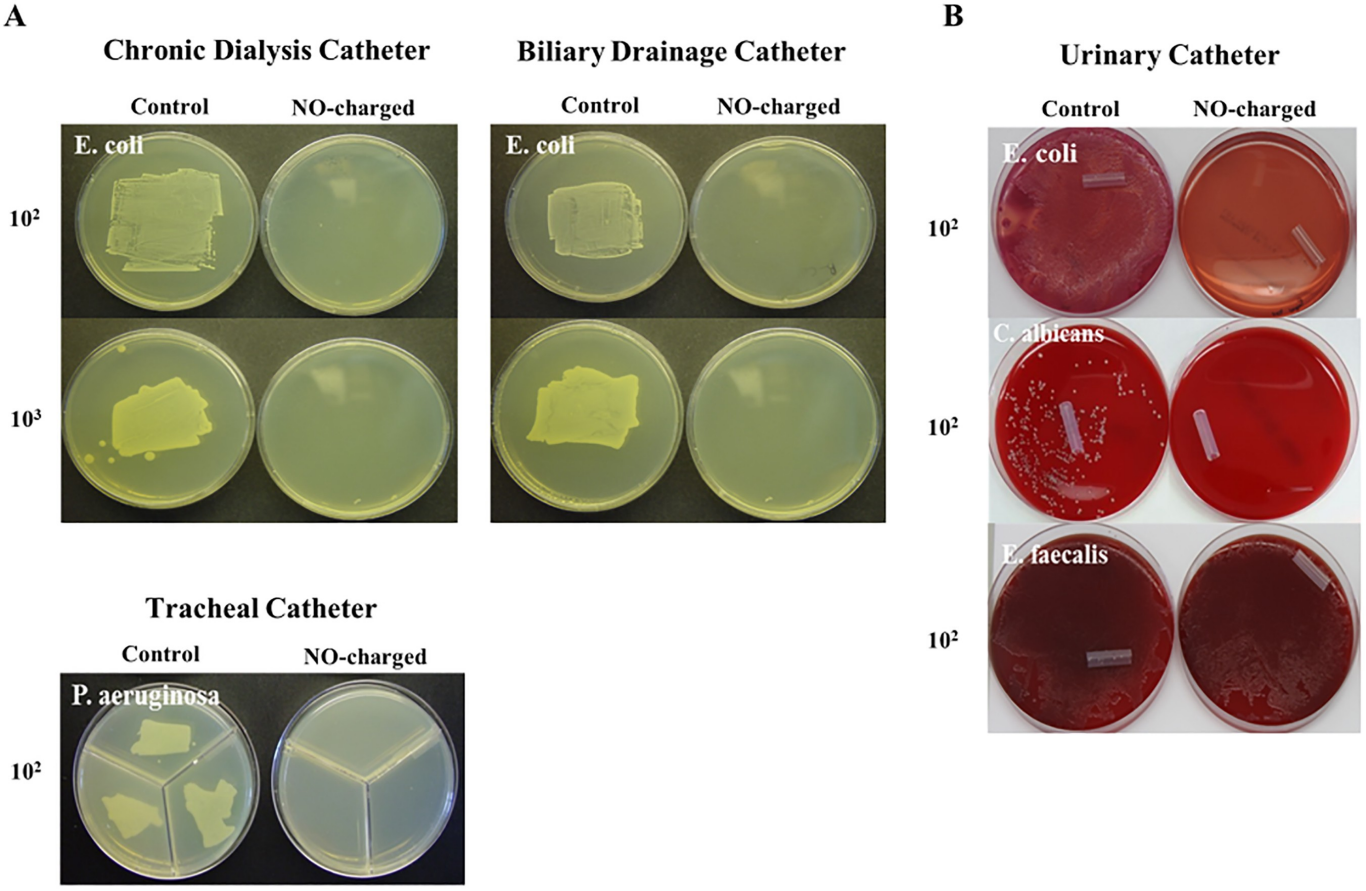
NO-charged catheters prevent microbial attachment. NO-charged and control dialysis, biliary, urinary and tracheal catheters (n = 3 per group) were divided into 2-cm sections and immersed in contaminated suspensions; Dialysis and biliary catheter sections in 10^2^ and 10^3^ CFU/ml of *E*. *coli*, urinary catheter sections in 10^2^ CFU/ml of *E*.*coli*, *C*. *albicans* or *E*. *faecalis* and tracheal catheter sections in 10^2^ CFU/ml of *P*. *aeruginosa*. Following 24-hour incubation bacterial and fungal attachment onto the catheters surfaces was qualitatively assessed after rolling of the catheter sections. (A) Comparison of *E*. *coli* attachment onto dialysis and biliary catheters rolled onto LB agar plates. (B) Comparison of *E*. *coli*, *C*. *albicans* and *E*. *faecalis* onto urinary catheters rolled onto blood agar plates (upper and lower pictures) or onto MacConkey agar plates (center picture). (C) Comparison of *P*. *aeruginosa* attachment onto tracheal catheters rolled onto MH agar plates.

To evaluate the ability of NO-charged catheters to prevent microbial attachment onto their surfaces, NO-charged and control catheter sections were exposed to suspensions contaminated with low concentration of different types of microbial cells; *E*. *coli*, *C*. *albicans*, *E*. *faecalis* and *P*. *aeruginosa*. Microbial attachment was evaluated after an incubation period of 24–48 hours. *E*. *coli*, *C*. *albicans*, *E*. *faecalis* and *P*. *aeruginosa* attached onto all corresponding control catheter sections. In contrast, *E*. *coli*, *C*. *albicans* and *P*. *aeruginosa* failed to attach onto NO-charged catheter sections, while *E*. *faecalis* attached onto NO-charged urinary catheter sections (Fig 3), albeit at lower affinity compared to non-charged catheters.

**Fig 4:**
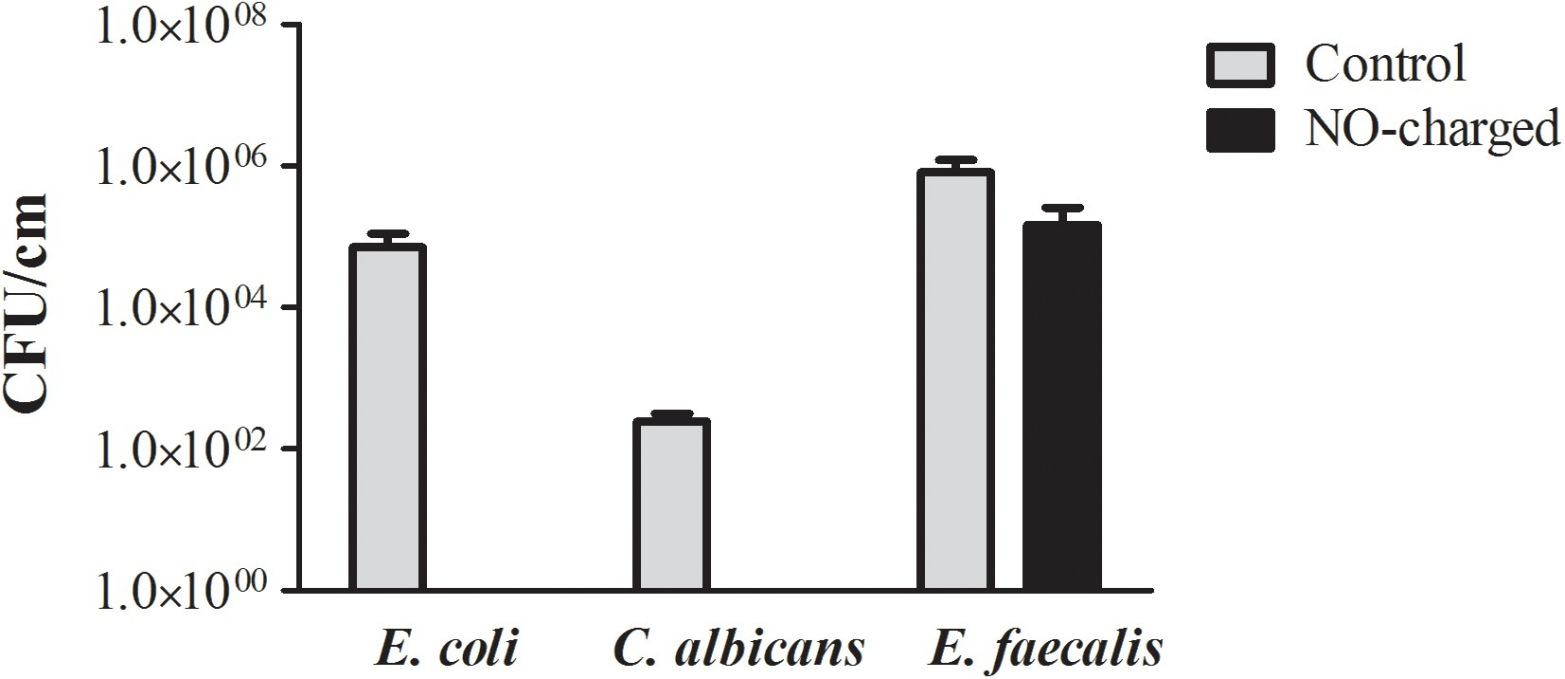
NO-charged catheters prevent/reduces microbial colonization. NO-charged and control urinary catheters (*n* = 3 per group) were divided into 2-cm sections and immersed in contaminated suspensions of 10^2^ CFU/ml of *E*.*coli*, *C*. *albicans* or *E*. *faecalis*. Following 24-hour incubation bacterial and fungal colonization per 1-cm of catheter section was quantitatively assessed after sonication of the catheter sections in fresh saline solution followed by vigorous vortex and plating onto MacConkey (*E*. *coli*) or blood agar plates (*E*. *faecalis* and *C*. *albicans*). Number of colonizing bacteria per 1-cm of catheter was determined the next day.

Quantitative evaluation of the number of colonizing microbial cells onto the surfaces of 1-cm sections of NO-charged and control urinary catheters was performed following an incubation period of 24 hours with *E*. *coli*, *C*. *albicans* and *E*. *faecalis* and sonication of the catheter sections in fresh saline solution to separate colonizing bacteria and fungi from their surfaces. As shown (Fig 4), *E*. *coli* and *C*. *albicans* colonization onto NO-charged urinary catheter sections was prevented; decreasing by approximately 5 logs and 2.5 logs compared to the control sections, respectively. *E*. *faecalis* colonization onto NO-charged urinary catheter sections decreased by 1 log compared to the control sections.

### NO-charged catheters prevent planktonic microbial growth

**Fig 5:**
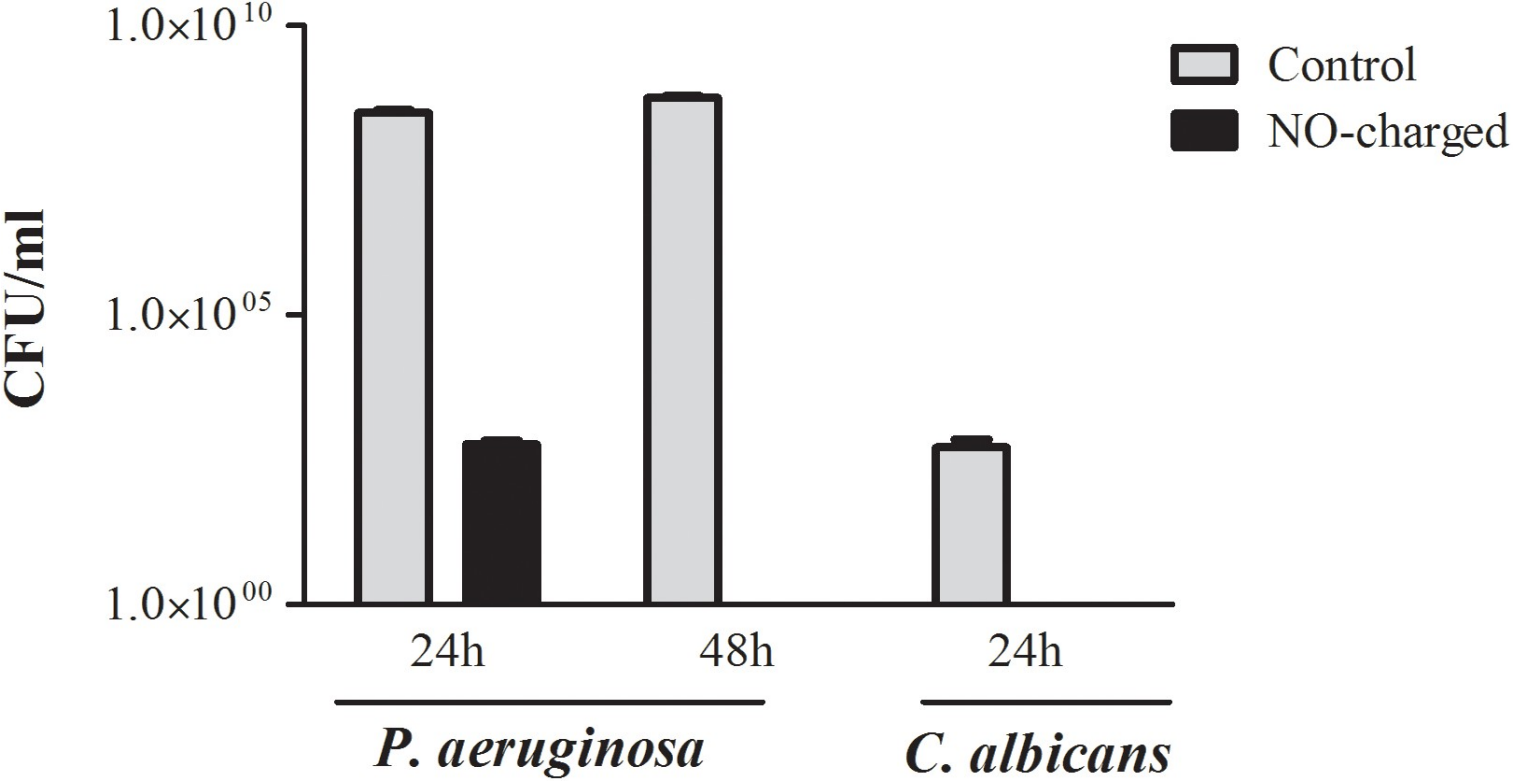
NO-charged catheters prevent planktonic microbial growth. Viable counts of *P*. *aeruginosa* and *C*. *albicans* (n = 3 for each group per time point) after immersion of NO-charged tracheal and urinary catheter sections in suspension containing 10^2^ CFU/ml of *P*. *aeruginosa* and *C*. *albicans* and incubation for 24–48 hours at 30°C and 37°C, respectively.

Although NO has a small radius of action in solution we have showed that it possesses antimicrobial activity against *E*. *coli* in solution [13–14]. To evaluate whether NO-charged catheters possesses anti-microbial activity against other bacterial and fungi we exposed tracheal catheter sections to 10^2^ CFU/ml of *P*. *aeruginosa* and urinary catheters to 10^2^ CFU/ml of *C*. *albicans* and tested bacterial content in suspensions after 24–48 hours of incubation. *P*. *aeruginosa* suspensions incubated with control catheter sections had 3.09×10^8^ CFU/ml and 5.6×10^8^ CFU/ml after 24 hours and 48 hours of incubation, respectively, while control *C*. *albicans* suspensions had 5.17×10^2^ CFU/ml after 24-hour incubation. In contrast, *P*. *aeruginosa* and *C*. *albicans* suspensions incubated with NO-charged catheters had 0 CFU/ml after 48 and 24 hours, respectively (Fig 5). Similarly, immersion of NO-charged dialysis and tracheal catheter sections in suspension containing 10^2^ CFU/ml of *E*. *coli* resulted in complete eradication of planktonic bacteria after 24 hours, while suspensions incubated with control catheters had up to 10^8^ CFU/ml of *E*. *coli* (data not shown).

### Safety and tolerability of NO-charged urinary catheters–phase 1 clinical study

#### Proportion of patients who completed the study and NO-related adverse events

Patients were enrolled to this study from the 22^nd^ of December, 2014 to the 30^th^ of March, 2015. Study was completed at 12^th^ of May, 2015 (end of follow-up period of the last patient). All patients from the NO-group successfully completed the study per protocol (Fig 1), without experiencing NO-related AE's (Table 1), while one patient from the CT-group failed to complete the study per protocol due to an SAE.

**Table 1:** AE's and SAE's recorded during the study.

	CT-Group		NO-Group		
	N	(%)	N	(%)	*P* Value
**No. of Patient**	6		6		
**No. of Patients with AE's**	2	(33%)	2	(33%)	1.000
**No. of Patients with SAE's**	1	(16.66%)	0	(0%)	1.000
**AE's associated with NO catheter**	N/A		0	(0%)	
**SAE's associated with NO catheter**	N/A		0	(0%)	
**AE's (Severity)**					
**Hematuria**	1 (Mild)	(16.66%)	1 (Mild)	(16.66%)	1.000
**Bleeding**	1 (Severe)	(16.66%)	0	(0%)	1.000
**Hyperkalemia**	0	(0%)	1(Mild)	(16.66%)	1.000
**SAE's**					
**Myocardial Infract**	1	(16.66%)	0	(0%)	1.000

Number and proportion of AE's and SAE's recorded during the study. Fisher's exact test was used for analyzing the difference in the number and the proportions of AE's and SAE's between both groups. *p* > 0.05, non-statistically significant difference. *p* < 0.05, statistically significant difference.

#### Physical examination

Since NO is associated with sustained penile erection and may cause local skin irritation and the formation of local skin edema and erythema to its surrounding tissue [15–20], a special emphasis was put on the genitourinary system in particular to the penis, scrotum or groin area during physical examinations performed at baseline, on a daily basis during hospitalization, at catheter removal day and at follow-up visit. No priapism or local irritation to the penis, scrotum or groin, were detected in both groups throughout the study. No other physical changes in the respiratory, cardiovascular or head, ears, eyes, nose and throat (HEENT) systems were observed in all patients from the NO-group, while the physical condition of one patient from the CT-group worsened during hospitalization due to myocardial infarct.

#### Vital signs and clinical laboratory evaluation

Vital signs measurements and biochemistry, hematology and coagulation tests were taken throughout the study and their values were compared between the CT-group and NO-group at the following time points: at baseline, at 1^st^ day of catheterization, at hospital discharge day, at catheter removal day and at follow-up visit. In addition, the change of each parameter value from baseline at each time point was assessed within each particular group.

Baseline results of parameters that may be associated with NO and their corresponding normal ranges are presented (Table 2). As shown, no statistically significant differences were detected at baseline between the groups, and all tests results of patients from both groups were within the accepted normal ranges.

Comparison results (CT vs NO group) of parameters that may be associated with NO throughout the study are presented at Table 3. Changes from baseline levels of these parameters within each group throughout the study are presented at Table 4.

**Table 2:** Patient Characteristics and baseline measurements.

**Characteristic**	**CT-Group**	**NO-Group**	***P* Value**	
**No. of patients**	6	6		
**Age (Yr)**	67 ± 3.39	68.33 ± 1.72	1.000	
**NO-related Parameters**	**Mean±SEM**	**Mean±SEM**	***P* Value**	**Normal range**
**Blood Pressure (mmHg)**	101.9 ± 7.79	95.26 ± 1.39	0.393	80–120
**Temperature (°C)**	36.75 ± 0.11	36.68 ± 0.060	0.685	36.5–37.2
**PT (Sec)**	11.33 ± 0.30	10.63 ± 0.21	0.169	9.5–13.5
**aPTT (Sec)**	32.65 ± 0.89	32.24 ± 1.62	0.792	24–40
**INR**	0.92 ± 0.02	0.88 ± 0.01	0.146	0.8–1.2
**Platelets (10**^**3**^**/μl)**	297.50 ± 81.69	213.20 ± 12.21	0.748	130–400
**Hemoglobin (g/dl)**	14.33 ± 0.53	14.83 ± 0.23	0.484	14–18
**WBC (10**^**3**^**/μl)**	6.51 ± 0.5	9.31 ± 1.28	0.093	4.8–10.8
**Neutrophils absolute (10**^**3**^**/μl)**	4.00 ± 0.5	4.08 ± 0.37	0.935	1.9–8.0
**CRP (mg/dl)**	0.31 ± 0.10	0.25 ± 0.12	0.753	0–0.5

Baseline levels vital signs and coagulation, hematological, and biochemical parameters that may be associated with NO. All tests were taken prior to radical prostatectomy surgery. Differences in parameters values were compared between the Control group [CT-group] and the Nitric Oxide group [NO-group] using The Non-parametric Mann-Whitney U-test. Accepted normal ranges are presented **Abbreviations**: PT–Prothrombin Time; aPTT–Activated Partial Thromboplastin Time; INR–International Normalized Ratio; WBC–White Blood Cells; CRP–C—reactive protein. *p* > 0.05, non-statistically significant difference. *p* < 0.05, statistically significant difference.

**Table 3:** Comparison of parameters associated with NO-releasing catheter–CT versus NO Group.

	CT-Group		NO-Group		
Parameters	N	Mean±SEM	N	Mean±SEM	*P* Value
**Blood Pressure (mmHg)**					
**1**^**st**^ **day of Catheterization**	6	96.47±6.206	6	90.68± 4.053	0.818
**Hospital Discharge**	6	104.40±2.861	6	99.70±3.553	0.394
**Catheter Removal**	6	106.8±5.455	6	102.40±5.495	0.521
**Follow-up**	6	106±3.801	6	98.08± 3.595	0.261
**Temperature (°C)**					
**1**^**st**^ **day of Catheterization**	6	36.86±0.086	6	36.81±0.164	0.470
**Hospital Discharge**	6	36.87±0.096	6	36.99±0.095	0.375
**Catheter Removal**	6	36.87±0.143	6	36.75±0.056	0.326
**Follow-up**	6	36.88±0.130	6	36.77±0.080	0.470
**PT (Sec)**					
**1**^**st**^ **day of Catheterization**	5	12.22±0.404	6	11.98±0.289	0.647
**Hospital Discharge**	6	11.98±0.547	6	11.40± 0.330	0.589
**Catheter Removal**	4	12.38±0.914	6	11.18±0.236	0.476
**Follow-up**	5	11.62±0.723	6	11.08±0.199	1.000
**Activated PTT (Sec)**					
**1**^**st**^ **day of Catheterization**	5	28.42±2.321	6	30.42±1.021	0.464
**Hospital Discharge**	6	28.98±2.114	6	32.28±1.031	0.240
**Catheter Removal**	4	30.70±0.930	6	34.15±1.411	0.114
**Follow-up**	5	31.30± 1.123	6	33.63±1.476	0.329
**INR**					
**1**^**st**^ **day of Catheterization**	5	0.99±0.032	6	0.99±0.020	0.855
**Hospital Discharge**	6	0.97±0.044	6	0.94±0.024	1.000
**Catheter Removal**	4	1.00±0.074	6	0.92±0.025	0.516
**Follow-up**	5	0.94±0.059	6	0.89±0.016	0.926
**Platelets (10**^**3**^**/μl)**					
**1**^**st**^ **day of Catheterization**	6	273.00±105.00	6	176.30±9.982	0.699
**Hospital Discharge**	6	273.50±90.330	6	193.50±13.40	0.818
**Catheter Removal**	6	352.70±74.450	6	321.30± 20.480	0.699
**Follow-up**	6	305.30±86.530	6	224.70±24.120	0.937
**Hemoglobin (g/dl)**					
**1**^**st**^ **day of Catheterization**	6	11.95±0.532	6	13.23±0.161	0.092
**Hospital Discharge**	6	12.72±0.711	6	13.37±0.437	0.521
**Catheter Removal**	6	13.17±0.761	6	14.47±0.531	0.394
**Follow-up**	6	13.28±0.789	6	14.43±0.438	0.240
**WBC (10**^3^/μl)					
**1**^**st**^ **day of Catheterization**	6	8.34±1.003	6	7.74±1.159	0.699
**Hospital Discharge**	6	8.62±0.796	6	7.438±1.038	0.394
**Catheter Removal**	6	8.09± 0.530	6	9.89±1.164	0.309
**Follow-up**	6	6.75±0.755	6	9.41±1.099	0.093
**Neutrophils (10**^3^/μl)					
**1**^**st**^ **day of Catheterization**	6	6.50±0.800	6	6.9±1.248	0.937
**Hospital Discharge**	6	6.45±0.712	6	5.567±0.382	0.394
**Catheter Removal**	6	5.68±0.711	6	6.517±0.624	0.394
**Follow-up**	6	4.63±0.847	6	5.017±0.240	0.297
**CRP (mg/dl)**					
**1**^**st**^ **day of Catheterization**	5	2.75±0.951	6	3.57±0.551	0.430
**Hospital Discharge**	6	7.86±3.797	6	6.96±1.003	0.485
**Catheter Removal**	6	4.82±3.838	6	0.87±0.251	0.818
**Follow-up**	6	4.65±3.865	6	0.42±0.154	0.240

Comparison of parameters values between the CT-group and the NO-group at 1^st^ day of catheterization, at hospital discharge, at catheter removal day and at follow-up visit. Non-parametric Mann-Whitney U-test for independent samples was applied for analyzing differences in parameters between the NO-group and CT-group at each time point. *p* > 0.05, non-statistically significant difference. *p* < 0.05, statistically significant difference.

**Table 4:** Parameters associated with NO-releasing catheter–change from baseline within groups.

	CT-Group				NO-Group			
**Parameters**	**N**	**Mean±SEM**	**% Change From Baseline**	**P value**	**N**	**Mean±SEM**	**% Change From Baseline**	**P value**
**Blood Pressure (mmHg)**								
**Baseline**	6	101.90±7.79			6	95.26±1.39		
**1**^**st**^ **day of Catheterization**	6	96.47±6.20	-5.35	0.598	6	90.68±4.05	-4.80	0.349
**Hospital Discharge**	6	104.40±2.86	2.40	0.709	6	99.70±3.55	4.66	0.265
**Catheter Removal**	6	106.8±5.45	4.74	0.364	6	102.40±5.49	7.44	0.317
**Follow-Up**	6	106±3.801	4.04	0.514	6	98.08±3.595	2.97	0.564
**Temperature (°C)**								
**Baseline**	6	36.75±0.11			6	36.68±0.06		
**1**^**st**^ **day of Catheterization**	6	36.86±0.09	0.29	0.405	6	36.81±0.16	0.35	0.46
**Hospital Discharge**	6	36.87±0.10	0.33	0.296	6	36.99±0.09	0.35	0.061
**Catheter Removal**	6	36.87±0.14	0.32	0.598	6	36.75±0.06	0.19	0.394
**Follow-Up**	6	36.88±0.130	0.36	0.299	6	36.77±0.080	0.23	0.434
**PT (Sec)**								
**Baseline**	6	11.33±0.307			6	10.63±0.219		
**1**^**st**^ **day of Catheterization**	5	12.22±0.404	9.18	0.019	6	11.98±0.289	12.70	< 0.001
**Hospital Discharge**	6	11.98±0.547	5.74	0.160	6	11.40±0.330	7.15	0.005
**Catheter Removal**	4	12.38±0.914	6.80	0.292	6	11.18±0.236	5.17	0.005
**Follow-Up**	5	11.62±0.723	3.88	0.402	6	11.08±0.199	4.23	0.048
**aPTT (Sec)**								
**Baseline**	6	32.65±0.896			5	32.24±1.627		
**1**^**st**^ **day of Catheterization**	5	28.42±2.321	-15.19	0.083	6	30.42±1.021	-6.33	0.035
**Hospital Discharge**	6	28.98±2.114	-11.21	0.150	6	32.28±1.031	-0.06	0.988
**Catheter Removal**	4	30.70±0.930	-5.73	0.048	6	34.15±1.411	5.65	0.088
**Follow-Up**	5	31.30±1.123	-2.14	0.441	6	33.63±1.476	4.96	0.013
**INR**								
**Baseline**	6	0.92±0.022			6	0.88±0.011		
**1**^**st**^ **day of Catheterization**	5	0.99±0.032	7.61	0.055	6	0.99±0.020	12.50	< 0.001
**Hospital Discharge**	6	0.97±0.044	4.35	0.233	6	0.94±0.024	6.82	0.008
**Catheter Removal**	4	1.00±0.074	6.52	0.309	6	0.92±0.025	4.55	0.101
**Follow-Up**	5	0.94±0.059	2.17	0.603	6	0.89±0.016	2.27	0.233
**Hemoglobin (g/dl)**								
**Baseline**	6	14.33±0.539			6	14.83±0.229		
**1**^**st**^ **day of Catheterization**	6	11.95±0.532	-16.61	< 0.001	6	13.23±0.161	-10.79	0.002
**Hospital Discharge**	6	12.72±0.711	-11.30	0.035	6	13.37±0.437	-9.91	0.016
**Catheter Removal**	6	13.17±0.766	-8.14	0.038	6	14.47±0.531	-2.49	0.517
**Follow-Up**	6	13.28±0.789	-7.33	0.075	6	14.43±0.438	-2.70	0.347
**WBC (10**^**3**^**/μl)**								
**Baseline**	6	6.51±0.497			6	9.307±1.283		
**1**^**st**^ **day of Catheterization**	6	8.34±1.003	28.11	0.042	6	7.74± 1.159	21.06	0.096
**Hospital Discharge**	6	8.62±0.796	32.41	0.093	6	7.438±1.038	7.95	0.416
**Catheter Removal**	6	8.09±0.530	24.27	0.125	6	9.89±1.164	21.06	0.091
**Follow-Up**	6	6.75±0.755	3.69	0.843	6	9.41±1.099	5.37	0.548
**Neutrophils (10**^**3**^**/μl)**								
**Baseline**	6	4.00±0.500			6	4.08± 0.368		
**1**^**st**^ **day of Catheterization**	6	6.50±0.800	62.50	0.015	6	6.90±1.248	69.12	0.041
**Hospital Discharge**	6	6.45±0.712	61.25	0.074	6	5.57±0.382	35.05	0.053
**Catheter Removal**	6	5.68±0.711	42.00	0.127	6	6.52±0.624	59.56	0.025
**Follow-Up**	6	4.63±0.847	15.75	0.599	6	5.02±0.240	22.79	0.084
**Platelets (10**^**3**^**/μl)**								
**Baseline**	6	297.5±81.7			6	213.2±12.21		
**1**^**st**^ **day of Catheterization**	6	273.0±105.0	-8.24	0.416	6	176.3±9.982	-17.27	0.005
**Hospital Discharge**	6	273.5±90.3	-8.07	0.179	6	193.50±13.4	-9.23	0.067
**Catheter Removal**	6	352.7±74.40	18.54	0.001	6	321.3±20.48	50.75	0.001
**Follow-Up**	6	305.3±86.50	2.63	0.679	6	224.7±24.12	5.39	0.619
**CRP (mg/dl)**								
**Baseline**	5	0.31±0.100			5	0.25±0.120		
**1**^**st**^ **day of Catheterization**	5	2.75±0.951	867.74	0.084	6	3.57±0.551	1236.00	0.005
**Hospital Discharge**	6	7.86±3.797	2841.94	0.114	6	6.96±1.003	2684.00	0.005
**Catheter Removal**	6	4.82±3.838	1748.39	0.304	6	0.87±0.251	292.00	0.047
**Follow-Up**	6	4.65±3.865	1693.55	0.321	6	0.42±0.154	88.00	0.205

Changes in parameters values from baseline levels during the study within both study groups were evaluated at the 1^st^ day of catheterization, at hospital discharge, at catheter removal day and at follow-up visit. Paired T-test was applied for analyzing changes from baseline levels. *p* > 0.05, non-statistically significant change from baseline. *p* < 0.05, statistically significant change from baseline.

No statistically significant differences in all parameters associated with NO were detected between the study groups throughout the study (Table 3).

No statistically significant changes from baseline in blood pressure or temperature were observed within each study group throughout the study (Table 4). Both CT and NO groups exhibited an increase from baseline levels in the coagulation parameters PT and INR, and a decrease in aPTT during hospitalization. However, in the NO-group the change from baseline of these parameters was statistically significant. In both groups the levels of all coagulation parameters were between the normal ranges.

Hemoglobin levels significantly decreased from baseline in both groups during hospitalization, where in the CT-group a significant decrease was detected also at catheter removal day. The mean values of hemoglobin in the CT-group during hospitalization and follow-up visit were lower than the normal ranges by 16.7% and 7.3%, respectively.

WBC and neutrophils significantly increased from baseline by 28.1% and 62.5%, in the CT-group at the 1^st^ day of catheterization, respectively. Similarly, an increase in WBC and neutrophils was also measured in the NO-group at the same corresponding time points. However, this increase was not statistically significant.

Platelets level decreased significantly from baseline levels by 17.3% in the NO-group at the 1^st^ day of catheterization. In contrast, at catheter removal day a significant increase from baseline levels was detected in both groups.

CRP levels significantly increased in the NO-group by 1236%, 2684%, and 292% in comparison to baseline levels during the 1^st^ day of catheterization, Hospital discharge, and catheter removal, respectively. CRP levels of the CT-group also increased during the same periods albeit without reaching statistical significance.

Excluding hemoglobin measurements, the mean values of all parameters during hospitalization, catheter removal and follow-up visits were within the normal ranges.

No significant differences in electrolytes, metabolites, renal function and liver function parameters were detected between the study groups (S1 Table). Changes from baseline levels were detected in several parameters in both groups, however all parameters at all time points were between the normal ranges (S2 Table).

### Validation of NO release from NO-charged urinary catheters

In order to confirm that the catheters used in the clinical study release NO, for each NO-charged catheter delivered for catheterization, an internal control measurement was taken to assess the levels of NO released after 24-hour exposure to fluids. NO was released from all urinary catheters at a concentration of between 1.75–2.8 ppm per catheter after 24 hours.

## Discussion

In this study we present pre-clinical data of the anti-infective properties of several types of NO-charged catheters *in-vitro*, and safety data from our phase 1 clinical study for the evaluation of the safety and tolerability of NO-charged urinary catheters in patients catheterized for 7–28 days.

In this study we chose to focus on the preventative capabilities of NO-charged catheters. Therefore the anti-infective properties of NO-charged catheters were tested against low concentration of bacteria and fungi which may attach onto the inner and outer surfaces of the catheters in small numbers during the first hours of catheterization. As shown, NO-charged catheters prevent gram-negative bacteria (*E*. *coli* and *P*. *aeruginosa*) and fungus (*C*. *albicans*) from attaching and colonizing onto their surfaces and exhibit some anti-microbial properties by eradicating small population of gram-negative bacteria and fungi in the surrounding media. This effect was more moderate in NO-charged urinary catheters tested against gram-positive bacteria (*E*. *faecalis*) where a 1 log reduction of in the number of colonizing bacteria was observed. In this case release of NO from the catheter had no effect on bacterial survival at the surrounding media.

Prevention/reduction of the colonization abilities of the microbial strains tested in this study are of clinical importance as biofilm-forming microbial cells are known to be play a significant role in the pathogenesis of catheter-associated HAI's and especially in UTI's and BSI's [23–25]. Most importantly, by reducing HAI's incidents cause by microbial pathogens it is reasonable to assume that NO-based charging technology may also reduce the amount of antibiotics given to patients, thus easing some of the concerns regarding excessive usage of antibiotics and the emergence of antibiotic-resistance microbial strains.

The anti-infective capabilities of NO-charged catheters demonstrated here were achieved against low concentrations of bacteria under a wide-range of NO concentrations released during the first few hours after immersion of the catheters in fluids. Although one may speculate that the anti-microbial protection exhibited here for tracheal, dialysis and biliary catheters may not last during the entire catheterization period since minimal levels of NO are released from these catheters after 24 hours, we believe that their ability to prevent early microbial colonization is an important step in preventing/reducing biofilm formation onto their surfaces and that this characteristic may have a dramatic impact on microbial susceptibility to various anti-microbial agents. Currently, we are working on increasing the duration of NO release from tracheal, dialysis and biliary catheters as already achieved for urinary catheters which release NO for a period of 14 days [13], so that they will better serve this important potential attribute.

The *in-vitro* data presented here were collected from three different experiments (Tracheal catheter study, chronic dialysis and biliary catheter, and urinary catheter study) that used several types of microbial cells and performed by different lab personal. The results presented in this study are in-line with data presented by Regev-Shoshani et al, which studied the NO-release profile from charged urinary catheters in urine and water, and their ability to prevent *E*. *coli* colonization and to elicit anti-microbial conditions *in-vitro* when exposed to low concentration of bacteria [13–14]. Taken together, NO-charged catheters characteristics demonstrated in this study and the consistency in the efficacy results supports the assumption that NO-charging technology might be effective in preventing/reducing catheter-associated HAI's and should be tested in clinical trials.

According to recent reports, UTI is a most common HAI; with Seventy-five percent of hospital-acquired UTI's are associated with catheterization [2]. Therefore, we chose to proceed with clinical development of NO-charged urinary catheters and test their safety and tolerability in catheterized patients.

NO-charged urinary catheters were found safe and well tolerated and met all primary endpoints; all patients catheterized with NO-charged urinary catheters successfully completed the study per protocol without experiencing AE's or SAE's associated with NO-charged catheters.

All physiological and coagulation (i.e. PT, aPTT and INR), hematological (i.e. Platelets, Hemoglobin, WBC, and Neutrophils) and biochemical (i.e. CRP) parameters that may be associated with NO-releasing catheters were compared between the groups throughout the study, and revealed no statistically significant differences.

Parameters values of CRP, PT, platelets, hemoglobin and WBC were changed in comparison to their baseline levels during the study. It seems that these changes are related to the radical prostatectomy surgery and to the medication given to the patients during the study, rather than to the inserted catheters. Indeed, post-operative elevation of CRP levels during hospitalization which is common after radical prostatectomy surgery [26], gradually decreased in both groups as time from catheterization proceeded. In addition, a gradual improvement of hemoglobin levels was also observed following an initial decrease observed during catheterization in both groups.

The decrease in blood coagulation parameters observed in both groups is primarily related to the anti-coagulative medication given to all patients prior to surgery to prevent venous thromboembolism, since all coagulation parameters improved in both groups as time from catheterization proceeded and when anti-coagulative medications were removed. Although, the decrease was markedly more evident in the NO-group, we assume that the effect of NO on blood coagulation is not clinically important since all parameters were between normal ranges at all time points, even though anti-coagulation medication was given. In addition, no bleeding episodes were documented in the NO-group throughout the study.

The safety data of NO-charged urinary catheters was expected since NO-charged urinary catheters release low concentration of NO. Although these concentrations are significantly lower (∼20-folds) than the NO levels approved by the Food and Drug administration (FDA) to treat infants suffering from persistent pulmonary hypertension of the newborn (PPHN) [27–28], they were found to elicit anti-microbial conditions in this and in previous studies [13–14].

## Conclusions

The pre-clinical data and safety profile of NO-charged urinary catheters in humans confirm that these catheters can be considered safe for future efficacy studies and places NO-based charging technology as a potential platform for the prevention of catheter-associated HAI's.

## Supporting information

S1 Table Comparison of electrolytes, metabolites, renal function and liver function parameters–CT versus NO Group. A comparison of parameters values between the CT group and the NO group at 1^st^ day of catheterization, at hospital discharge, at catheter removal day and at follow-up visit. Non-parametric Mann-Whitney U-test for independent samples was applied for analyzing differences in parameters between the NO group and CT group at each time point. *p* < 0.05, statistically significant difference; *p* > 0.05, Non-statistically significant difference. **Abbreviations**: ALP–Alkaline Phosphatase; AST–Aspartate Aminotransferase; ALT—Alanine Aminotransferase; GGT–Gamma-Glutamyl Transferase.
(DOCX)

S2 Table Electrolytes, metabolites, renal function and liver function parameters–change from baseline within groups. Changes in parameters values from baseline levels during the study within both study groups were evaluated at the 1st day of catheterization, at hospital discharge, at catheter removal day and at follow-up visit. Paired T-test was applied for analyzing changes from baseline levels. *p* < 0.05, statistically significant change from baseline; *p* > 0.05, Non-statistically significant change from baseline.
(DOCX)

S1 File Individual data. Individual participant data for all parameters evaluated during the study.
(XLSX)

S2 File Clinical trial protocol. (PDF)

S3 File Trend statement checklist. (PDF)
