# Supplementary material for: Nitric oxide charged catheters as a potential strategy for prevention of hospital acquired infections
Source: PLoS One. 2017 Apr 14;12(4):e0174443. doi: 10.1371/journal.pone.0174443 (PMC5391919; doi:10.1371/journal.pone.0174443)
Supplement: S1 Table — A comparison of parameters values between the CT group and the NO group at 1st day of catheterization, at hospital discharge, at catheter removal day and at follow-up visit. Non-parametric Mann-Whitney U-test for independent samples was applied for analyzing differences in parameters between the NO group and CT group at each time point. p < 0.05, statistically significant difference; p > 0.05, Non-statistically significant difference. Abbreviations: ALP–Alkaline Phosphatase; AST–Aspartate Aminotransferase; ALT—Alanine Aminotransferase; GGT–Gamma-Glutamyl Transferase. (DOCX) [file pone.0174443.s001.docx]

**S Table 1. Comparison of electrolytes, metabolites, renal function and liver function parameters – CT versus NO Group**

|  | **CT-Group** | | **NO-Group** | |  |
| --- | --- | --- | --- | --- | --- |
| **Parameters** | **N** | **Mean±SEM** | **N** | **Mean±SEM** | ***P* Value** |
| **Heart Rate (Beats/min)** |  |  |  |  |  |
| **1^st^ day of Catheterization** | 6 | 75.33±3.068 | 6 | 71.00±5.875 | 0.310 |
| **Hospital Discharge** | 6 | 82.36±3.731 | 6 | 73.67±5.134 | 0.199 |
| **Catheter Removal** | 6 | 81.67±5.954 | 6 | 75.17±4.700 | 0.521 |
| **Follow-Up** | 6 | 81.00±4.933 | 6 | 72.17±4.929 | 0.199 |
| **Urea (mg/dl)** |  |  |  |  |  |
| **1^st^ day of Catheterization** | 6 | 28.33±3.676 | 6 | 22.5± 2.262 | 0.261 |
| **Hospital Discharge** | 6 | 28.83±3.229 | 6 | 20.83±1.99) | 0.092 |
| **Catheter Removal** | 6 | 34.33±3.556 | 6 | 29.33±2.348 | 0.518 |
| **Follow-Up** | 6 | 36.00±3.847 | 6 | 38.00±2.295 | 0.336 |
| **Creatinine (mg/dl)** |  |  |  |  |  |
| **1^st^ day of Catheterization** | 6 | 0.94±0.958 | 6 | 0.87±0.081 | 0.588 |
| **Hospital Discharge** | 6 | 0.93±0.069 | 6 | 0.88±0.084 | 0.699 |
| **Catheter Removal** | 6 | 0.98±0.080 | 6 | 0.92±0.092 | 0.699 |
| **Follow-Up** | 6 | 0.97±0.094 | 6 | 0.94±0.086 | 0.809 |
| **Bilirubin (mg/dl)** |  |  |  |  |  |
| **1^st^ day of Catheterization** | 5 | 0.51±0.140 | 6 | 0.72±0.140 | 0.247 |
| **Hospital Discharge** | 6 | 0.64±0.150 | 6 | 0.69±0.100 | 0.699 |
| **Catheter Removal** | 6 | 0.57±0.200 | 6 | 0.43±0.047 | 0.589 |
| **Follow-Up** | 6 | 0.55±0.140 | 6 | 0.55±0.098 | 0.630 |
| **Sodium (mEq/L)** |  |  |  |  |  |
| **1^s^t day of Catheterization** | 6 | 139.00±1.400 | 6 | 141.00±0.70 | 0.255 |
| **Hospital Discharge** | 6 | 140.00±0.500 | 6 | 140.00±0.80 | 0.507 |
| **Catheter Removal** | 6 | 140.00±0.760 | 6 | 141.00±0.68 | 0.743 |
| **Follow-Up** | 6 | 141.00±0.920 | 6 | 141.00±0.71 | 0.624 |
| **Potassium (mEq/L)** |  |  |  |  |  |
| **1^st^ day of Catheterization** | 6 | 4.15±0.180 | 6 | 4.21±0.114 | 1.000 |
| **Hospital Discharge** | 6 | 4.18±0.117 | 6 | 4.30±0.115 | 0.626 |
| **Catheter Removal** | 6 | 4.43±0.209 | 6 | 4.76±0.276 | 0.374 |
| **Follow-Up** | 6 | 4.10±0.193 | 6 | 4.73± 0.181 | 0.054 |
| **ALP (U/L)** |  |  |  |  |  |
| **1^st^ day of Catheterization** | 5 | 60.60±6.508 | 6 | 64.67±6.907 | 0.537 |
| **Hospital Discharge** | 6 | 69.67±3.593 | 6 | 65.17±7.002 | 0.818 |
| **Catheter Removal** | 6 | 80.83±5.154 | 6 | 79.00±7.724 | 0.936 |
| **Follow-Up** | 6 | 79.33±8.365 | 6 | 80.67±8.204 | 1.000 |
| **AST (U/L)** |  |  |  |  |  |
| **1^st^ day of Catheterization** | 5 | 16.20±1.772 | 6 | 16.83±1.797 | 0.927 |
| **Hospital Discharge** | 6 | 27.67±4.800 | 6 | 17.33±0.246 | 0.148 |
| **Catheter Removal** | 6 | 23.67±3.148 | 6 | 19.50±1.945 | 0.469 |
| **Follow-Up** | 6 | 21.67±3.051 | 6 | 19.50±2.262 | 0.630 |
| **ALT (U/L)** |  |  |  |  |  |
| **1^st^ day of Catheterization** | 5 | 16.80±3.105 | 6 | 14.83±1.376 | 0.784 |
| **Hospital Discharge** | 6 | 24.50±7.496 | 6 | 14.83±1.222 | 0.378 |
| **Catheter Removal** | 6 | 30.00±10.390 | 6 | 20.33±2.011 | 1.000 |
| **Follow-Up** | 6 | 24.00±6.807 | 6 | 18.83±2.982 | 0.809 |
| **GGT (U/L)** |  |  |  |  |  |
| **1^st^ day of Catheterization** | 6 | 20.40±5.036 | 6 | 14.67±2.716 | 0.234 |
| **Hospital Discharge** | 6 | 49.33±23.586 | 6 | 16.83±2.469 | 0.262 |
| **Catheter Removal** | 6 | 53.67±20.140 | 6 | 24.17±3.781 | 0.126 |
| **Follow-Up** | 6 | 38.17±10.230 | 6 | 20.00±3.502 | 0.142 |
| **Glucose (mg/dl)** |  |  |  |  |  |
| **1^st^ day of Catheterization** | 6 | 109.50±13.440 | 6 | 105.5±5.486 | 0.873 |
| **Hospital Discharge** | 6 | 102.80±5.263 | 6 | 98.17±4.757 | 0.310 |
| **Catheter Removal** | 6 | 101.20±6.843 | 6 | 96.50±5.328 | 0.818 |
| **Follow-Up** | 6 | 106.30±7.246 | 6 | 107.3±12.92 | 0.809 |
| **Uric Acid (mg/dl)** |  |  |  |  |  |
| **1^st^ day of Catheterization** | 5 | 4.84±0.537 | 6 | 4.13±0.403 | 0.464 |
| **Hospital Discharge** | 6 | 5.38±0.457 | 6 | 4.20±0.338 | 0.078 |
| **Catheter Removal** | 6 | 5.65±0.445 | 6 | 4.76±0.431 | 0.240 |
| **Follow-Up** | 6 | 5.91±0.484 | 6 | 5.01±0.382 | 0.240 |

A comparison of parameters values between the CT group and the NO group at 1^st^ day of catheterization, at hospital discharge, at catheter removal day and at follow-up visit. Non-parametric Mann-Whitney U-test for independent samples was applied for analyzing differences in parameters between the NO group and CT group at each time point. *p* < 0.05, statistically significant difference; *p* > 0.05, Non-statistically significant difference. **Abbreviations**: ALP – Alkaline Phosphatase; AST – Aspartate Aminotransferase; ALT - Alanine Aminotransferase; GGT – Gamma-Glutamyl Transferase.
