# Supplementary material for: Nitric oxide charged catheters as a potential strategy for prevention of hospital acquired infections
Source: PLoS One. 2017 Apr 14;12(4):e0174443. doi: 10.1371/journal.pone.0174443 (PMC5391919; doi:10.1371/journal.pone.0174443)
Supplement: S2 Table — Changes in parameters values from baseline levels during the study within both study groups were evaluated at the 1st day of catheterization, at hospital discharge, at catheter removal day and at follow-up visit. Paired T-test was applied for analyzing changes from baseline levels. p < 0.05, statistically significant change from baseline; p > 0.05, Non-statistically significant change from baseline. (DOCX) [file pone.0174443.s002.docx]

**S2 Table. Electrolytes, metabolites, renal function and liver function parameters – change from baseline within groups**

|  | **CT-Group** | | | | **NO-Group** | | | | |
| --- | --- | --- | --- | --- | --- | --- | --- | --- | --- |
| **Parameters** | **N** | **Mean±SEM** | **% Change From Baseline** | **P value** | **N** | **Mean±SEM** | **% Change From Baseline** | | **P value** |
| **Heart Rate (Beats/min)** |  |  |  |  |  |  |  |  | |
| **Baseline** | 6 | 83.00±5.972 |  |  | 6 | 68.33±5.897 |  |  | |
| **1^st^ day of Catheterization** | 6 | 75.33±3.068 | -9.23 | 0.284 | 6 | 71.00 (5.875) | 3.89 | 0.461 | |
| **Hospital Discharge** | 6 | 82.36±3.731 | -0.77 | 0.929 | 6 | 73.67 (5.134) | 7.80 | 0.235 | |
| **Catheter Removal** | 6 | 81.67±5.954 | -1.60 | 0.810 | 6 | 75.17 (4.700) | 10.00 | 0.205 | |
| **Follow-Up** | 6 | 81.00±4.933 | -2.41 | 0.713 | 6 | 72.17 (4.929) | 5.61 | 0.521 | |
| **Urea (mg/dl)** |  |  |  |  |  |  |  |  | |
| **Baseline** | 6 | 35.83±4.672 |  |  | 6 | 36.17 (2.358) |  |  | |
| **1^st^ day of Catheterization** | 6 | 28.33±3.676 | -20.93 | 0.047 | 6 | 22.5 (2.262) | -37.79 | 0.007 | |
| **Hospital Discharge** | 6 | 28.83±3.229 | -19.54 | 0.089 | 6 | 20.83 (1.990) | -42.38 | 0.001 | |
| **Catheter Removal** | 6 | 34.33±3.556 | -4.19 | 0.630 | 6 | 29.33 (2.348) | -18.88 | 0.075 | |
| **Follow-Up** | 6 | 36.00±3.847 | 0.45 | 0.953 | 6 | 38.00 (2.295) | 5.06 | 0.614 | |
| **Creatinine (mg/dl)** |  |  |  |  |  |  |  |  | |
| **Baseline** | 6 | 1.00±0.068 |  |  | 6 | 1.00 (0.085) |  |  | |
| **1^st^ day of Catheterization** | 6 | 0.94±0.958 | -7.00 | 0.460 | 6 | 0.87 (0.081) | -13.00 | 0.112 | |
| **Hospital Discharge** | 6 | 0.93±0.069 | -8.00 | 0.209 | 6 | 0.88 (0.084) | -12.00 | 0.069 | |
| **Catheter Removal** | 6 | 0.98±0.080 | -3.00 | 0.694 | 6 | 0.92 (0.092) | -7.00 | 0.204 | |
| **Follow-Up** | 6 | 0.97±0.094 | -4.00 | 0.582 | 6 | 0.94 (0.086) | -6.00 | 0.124 | |
| **Bilirubin (mg/dl)** |  |  |  |  |  |  |  |  | |
| **Baseline** | 6 | 1.60±1.000 |  |  | 6 | 0.52 (0.110) |  |  | |
| **1^st^ day of Catheterization** | 5 | 0.51±0.140 | -80.00 | 0.355 | 6 | 0.72 (0.140) | 36.54 | 0.242 | |
| **Hospital Discharge** | 6 | 0.64±0.150 | -59.38 | 0.405 | 6 | 0.69 (0.100) | 30.77 | 0.107 | |
| **Catheter Removal** | 6 | 0.57±0.200 | -63.75 | 0.386 | 6 | 0.43 (0.047) | -17.31 | 0.374 | |
| **Follow-Up** | 6 | 0.55±0.140 | -65.00 | 0.365 | 6 | 0.55 (0.098) | 5.77 | 0.667 | |
| **Sodium (mEq/L)** |  |  |  |  |  |  |  |  | |
| **Baseline** | 6 | 140.00±0.88 |  |  | 6 | 140.00 (0.870) |  |  | |
| **1^st^ day of Catheterization** | 6 | 139.00±1.40 | -0.83 | 0.560 | 6 | 141.00 (0.710) | 0.59 | 0.363 | |
| **Hospital Discharge** | 6 | 140.00±0.50 | -0.11 | 0.894 | 6 | 140.00 (0.870) |  | N/A | |
| **Catheter Removal** | 6 | 140.00±0.76 | 0.47 | 0.655 | 6 | 141.00 (0.680) | 0.83 | 0.287 | |
| **Follow-Up** | 6 | 141.00±0.92 | 0.71 | 0.111 | 6 | 141.00 (0.710) | 1.07 | 0.312 | |
| **Potassium (mEq/L)** |  |  |  |  |  |  |  |  | |
| **Baseline** | 5 | 4.50±0.182 |  |  | 6 | 4.65 (0.246) |  |  | |
| **1^st^ day of Catheterization** | 6 | 4.15±0.180 | -10.67 | 0.090 | 6 | 4.21 (0.114) | -9.25 | 0.061 | |
| **Hospital Discharge** | 6 | 4.18±0.117 | -6.67 | 0.119 | 6 | 4.30 (0.115) | -7.53 | 0.070 | |
| **Catheter Removal** | 6 | 4.43±0.209 | 0.00 | 1.000 | 6 | 4.76 (0.276) | 2.37 | 0.785 | |
| **Follow-Up** | 6 | 4.10±0.193 | -8.89 | 0.138 | 6 | 4.73 (0.181) | 1.72 | 0.813 | |
| **ALP (U/L)** |  |  |  |  |  |  |  |  | |
| **Baseline** | 6 | 84.67±8.094 |  |  | 6 | 78.33 (7.919) |  |  | |
| **1^st^ day of Catheterization** | 5 | 60.60±6.508 | -23.62 | 0.001 | 6 | 64.67 (6.907) | -17.45 | 0.013 | |
| **Hospital Discharge** | 6 | 69.67±3.593 | -17.72 | 0.173 | 6 | 65.17 (7.002) | -16.81 | < 0.001 | |
| **Catheter Removal** | 6 | 80.83±5.154 | -4.52 | 0.702 | 6 | 79.00 (7.724) | 0.84 | 0.877 | |
| **Follow-Up** | 6 | 79.33±8.365 | -6.30 | 0.571 | 6 | 80.67 (8.204) | 2.97 | 0.116 | |
| **AST (U/L)** |  |  |  |  |  |  |  |  | |
| **Baseline** | 6 | 28.50±6.412 |  |  | 6 | 26.0 (4.65) |  |  | |
| **1^st^ day of Catheterization** | 5 | 16.20±1.772 | -21.75 | 0.002 | 6 | 16.83 (1.80) | -35.23 | 0.055 | |
| **Hospital Discharge** | 6 | 27.67±4.800 | -2.91 | 0.919 | 6 | 17.33 (2.25) | -33.35 | 0.088 | |
| **Catheter Removal** | 6 | 23.67±3.148 | -16.95 | 0.466 | 6 | 19.50 (1.95) | -25.00 | 0.224 | |
| **Follow-Up** | 6 | 21.67±3.051 | -23.96 | 0.280 | 6 | 19.50 (2.26) | -25.00 | 0.284 | |
| **ALT (U/L)** |  |  |  |  |  |  |  |  | |
| **Baseline** | 6 | 28.17±4.672 |  |  | 6 | 22.50 (3.128) |  |  | |
| **1^st^ day of Catheterization** | 5 | 16.80±3.105 | -29.82 | 0.003 | 6 | 14.83 (1.376) | -34.04 | 0.001 | |
| **Hospital Discharge** | 6 | 24.50±7.496 | -12.99 | 0.580 | 6 | 14.83 (1.222) | -34.04 | 0.045 | |
| **Catheter Removal** | 6 | 30.00±10.39 | 6.50 | 0.844 | 6 | 20.33 (2.011) | -9.60 | 0.415 | |
| **Follow-Up** | 6 | 24.00±6.807 | -14.77 | 0.486 | 6 | 18.83 (2.982) | -16.27 | 0.500 | |
| **GGT (U/L)** |  |  |  |  |  |  |  |  | |
| **Baseline** | 5 | 34.33±7.680 |  |  | 5 | 18.00 (3.207) |  |  | |
| **1^st^ day of Catheterization** | 6 | 20.40±5.036 | -27.96 | 0.027 | 6 | 14.67 (2.716) | -18.89 | 0.018 | |
| **Hospital Discharge** | 6 | 49.33±23.58 | 43.69 | 0.428 | 6 | 16.83 (2.469) | -10.00 | 0.088 | |
| **Catheter Removal** | 6 | 53.67±20.14 | 56.31 | 0.227 | 6 | 24.17 (3.781) | 32.22 | 0.056 | |
| **Follow-Up** | 6 | 38.17±10.23 | 11.16 | 0.229 | 6 | 20.00 (3.502) | 14.44 | 0.256 | |
| **Glucose (mg/dl)** |  |  |  |  |  |  |  |  | |
| **Baseline** | 6 | 91.00±4.789 |  |  | 6 | 98.67 (4.047) |  |  | |
| **1^st^ day of Catheterization** | 6 | 109.5±13.44 | 20.33 | 0.314 | 6 | 105.50 (5.488) | 6.92 | 0.250 | |
| **Hospital Discharge** | 6 | 102.80±5.263 | 13.00 | 0.182 | 6 | 98.17 (4.757) | -0.51 | 0.950 | |
| **Catheter Removal** | 6 | 101.2±6.843 | 11.18 | 0.327 | 6 | 96.50 (5.328) | -2.19 | 0.734 | |
| **Follow-Up** | 6 | 106.3±7.246 | 16.85 | 0.086 | 6 | 107.30 (12.920) | 8.78 | 0.571 | |
| **Uric Acid (mg/dl)** |  |  |  |  |  |  |  |  | |
| **Baseline** | 6 | 6.23±0.498 |  |  | 6 | 5.36 (0.414) |  |  | |
| **1^st^ day of Catheterization** | 5 | 4.84±0.537 | -21.19 | 0.005 | 6 | 4.13 (0.403) | -22.95 | <0.001 | |
| **Hospital Discharge** | 6 | 5.38±0.457 | -13.64 | 0.086 | 6 | 4.20 (0.338) | -21.64 | <0.001 | |
| **Catheter Removal** | 6 | 5.65±0.445 | -9.31 | 0.206 | 6 | 4.76 (0.431) | -11.19 | 0.074 | |
| **Follow-Up** | 6 | 5.91±0.484 | -4.98 | 0.547 | 6 | 5.01 (0.382) | -6.53 | <0.01 | |

Changes in parameters values from baseline levels during the study within both study groups were evaluated at the 1^st^ day of catheterization, at hospital discharge, at catheter removal day and at follow-up visit. Paired T-test was applied for analyzing changes from baseline levels. *p* < 0.05, statistically significant change from baseline; *p* > 0.05, Non-statistically significant change from baseline.
