# Supplementary material for: Nitric oxide charged catheters as a potential strategy for prevention of hospital acquired infections
Source: PLoS One. 2017 Apr 14;12(4):e0174443. doi: 10.1371/journal.pone.0174443 (PMC5391919; doi:10.1371/journal.pone.0174443)
Supplement: S2 File — (PDF) [file pone.0174443.s004.pdf]

Study Title: Prospective, Phase I, Single-Center, Evaluation of the Safety and Tolerability of Nitric Oxide Impregnated Urinary Catheters in Patients Undergoing Radical Prostatectomy

Protocol No.: ENOX_PHASE_I_1.1

Clinical Phase: I

Protocol Version Number 1.2 & Date: 23.03.15

Principal Investigator: Dr. David Margel

Department of Urology, Beilinson Medical Center, Israel

Cell: +972-50-7890053

Tel: +972-3-9377920

Fax: +972-3-9377902

E. Mail: sdmargel@gmail.com

Sponsor: ENOX Ltd

Address: Kibbutz Bet Alfa, Israel

Cell: +972-52-2854963

Tel: +972-4-6533552

Fax: + 972-4-6533471

E. Mail: dan.sadeh@enoxbiopharma.com

This clinical study will be conducted in accordance with the ENOX's Standard Operating Procedures (SOPs), current Good Clinical Practice (GCP), the provisions of ICH (International Conference on Harmonization) Guidelines

CONFIDENTIAL

The information in this document is considered privileged and confidential, and may not be disclosed to others except to the extent necessary to obtain Institutional Review Board/Ethics Committee approval, informed consent and the approval of local regulatory authorities as required by local law.

Protocol Review & Approval

PROTOCOL: ENOX_PHASE_I_1.1

Protocol Version: 1.2 23/03/2015

Prospective, Phase I, Single-Center, Evaluation of the Safety and Tolerability of Nitric Oxide Impregnated Urinary Catheters in Patients Undergoing Radical Prostatectomy

The undersigned have reviewed the format and content of this protocol and have approved Protocol No. ENOX_PHASE_I_1.1 for issuance.

**Position**	**Print name**	**Date**	**Signature**
Principal Investigator	Dr. David Margel		
ENOX Ltd. – Sponsor	Danny Sadeh		

INVESTIGATOR SIGNATURE SHEET

I have read the protocol and agree that it contains all the necessary details for performing the trial.

I will provide copies of the protocol and of the pre-clinical information on the test article, which was furnished to me by the sponsor, to all members of the study team for whom I am responsible and who participate in the trial. I will discuss this material with them to ensure that they are fully informed regarding the test article and the conduct of the trial.

Once the protocol has been approved by the IRB, I will not modify this protocol without obtaining the prior approval of the Sponsor and of the IRB. I will submit the protocol modifications and/or any informed consent modifications to the sponsor and the IRB, and approval will be obtained before any modifications are implemented.

I understand the protocol and will work according to it, the principles of Good Clinical Practice (current ICH guidelines), and the Declaration of Helsinki (1964) including all amendments up to and including the Seoul revision (2008).

Print Name	Investigator's Signature	Date

TABLE OF CONTENTS

1. SYNOPSIS 7
2. LIST OF ABBREVEATIONS 12
3. INTRODUCTION 13
3.1 Background 13
3.2 Study Hypothesis 17
3.3 Summary 18
4. STUDY OBJECTIVES 19
4.1 Primary Objective 19
4.2 Observational 19
5. STUDY END POINTS 20
5.1 Primary End Points 20
5.2 Observational 20
5.3 Safety and Tolaribility 20
5.3.1 Vital Signs Measurments 20
5.3.2 Laboratory Tests: Blood & Urine 21
6. STUDY DESIGN 22
6.1 Overview And Plan 22
6.2 Study Design and Schedule Visits (Visit 1 - Visit 5) 23
7. STUDY POPULATION 24
7.1 Number of Patients 24
7.2 Inclusion Criteria 24
7.3 Exlusion Criteria 25
7.4 End of Treatment Assesment 25
7.5 Early Treatment Discontinuation 25
7.5.1 Criteria For Treatment Discontinuation 25
7.5.2 Criteria For Early Study Withdrawal 26
7.5.3 Criteria For Study Stop 26
7.5.4 Replacement of Withdrawal patients 26
8. STUDY CONDUCT DETAILS DTUDY PLAN 27
8.1 Screening & Enrolments Procedures 27
8.1.1 Screening & Enrolment - Visit 1 (day 0) 27
8.2 Randomization 27
8.3 Study Treatment 28

8.3.1 Operation and Catheterization - Visit 2 (day 1)..28

8.3.2 Hospitalization - Visit 2 continuation (day 2 - discharge day)..................................28

8.3.3 Catheter Removal - Visit 3 (day 7-21±7)..28

8.4 Follow-up Visits...29

8.4.1 Telephone Call - Visit 4 (day 21±3) 29

8.4.2 Follow-up Visit - Visit 5 (day 30 - 45)..29

8.5 Unscheduled Visits..29

8.6 Termination/Study Early Discontinuation 29

8.7 Emergency Code Breaking..30

9. DESCRIPTION OF INVESTIGATIONAL MEDICINAL PRODUCTS/STUDY 31

9.1 Foley Catheter Embedded With Nitric Oxide...31

9.2 Impregnating Device..31

9.3 Impregnation Process 31

9.4 Sterilization/Validation...32

10. RISK ANALYSIS..33

11. ASSESMENT METHODS 34

11.1 Observational 34

11.2 Medical/Clinical Assessment...34

11.3 Clinical Laboratory Assessment/Evaluation 35

11.3.1 Blood Tests...35

11.3.2 Bacteriological Tests 35

11.4 Safety Parameters 36

11.4.1 Adverse Events 36

11.4.2 Potential Adverse Events Related to NO-impregnated Catheters 36

11.4.3 Safety Laboratory Evaluations..37

12. SAFETY...38

13. STATISTICAL METHODOLOGY 41

13.1 Sample Size Rationale..42

13.2 Randomization 42

13.3 Patient Cohort...42

13.4 Comparability of Treatment Groups at Baseline 43

14. REGULATORY AND ETHICAL ISSUES 44

14.1 Compliance With Regulations Applicabale to Clinical Trials....................................44

14.2 Informed Consent 44

14.3 Institutional Review Board (IRB)..44
14.4 Protocol Amendments 45
14.5 Decleration of The End of The Clinical Trial..45
14.6 Liability and Insurence 45
15. DOCUMENTATION..46
15.1 Study File and Site Documents 46
15.2 Study documents supplied by the Sponsor 46
15.3 Maintenance and Retention of Records 47
16. DATA HANDELING...48
16.1 Data Collection via Paper CRF 48
16.2 Data Entry 48
16.3 Medical Information Coding 49
16.4 Data Validation 49
16.4.1 Data Correction...49
16.4.2 Data Queries 50
16.4.3 Data Extract 50
16.4.4 Source Documents...50
16.4.5 Additional Documents and Records 50
17. QUALITY ASSURENCE...51
17.1 Good Clinical Practice...51
17.2 Good Laboratory Standards...51
17.3 Quality Assurance Program...51
17.4 Regulatory Inspections 51
18. STUDY MONITORING 52
18.1 Monitors/CRAs and Monitoring Visits...52
18.2 Primary Source Documents 52
19. USE OF INFORMATION AND PUBLICATION..54
20. STUDY PERSONAL 55
20.1 Investigative Site 55
20.2 Data Management and Biostatistics..55
20.3 Monitor/Clinical Research Associate 55
21. REFERENCE LIST...56
22. APPENDICES 58

1. SYNOPSIS

Protocol number	ENOX_PHASE_I_1.1
**Study Title:**	Prospective, Phase I, Single-Center, Evaluation of the Safety and Tolerability of Nitric Oxide Impregnated Urinary Catheters in Patients Undergoing Radical Prostatectomy
**Number of centers:**	1
**Center Name:**	Beilinson Medical Center, Department of Urology
**Clinical Phase:**	1
**Study Duration:**	The duration of the study for each patient is 30-45 days (treatment + follow up)<br>The expected duration of the study is approximately 6 months (screening, enrollment and data analysis until final study report)
**Study Population:**	Phase I: 12 patients (male), over the age of 18, undergoing radical prostatectomy
**Study Objectives:**	**Primary Objectives:**<br><br>**Safety and tolerability**<br>• Assess the safety of NO-Impregnated Foley catheters indwelling for 7-21±7 days in patients undergoing radical prostatectomy<br>• Assess the tolerability of NO-impregnated Foley catheters indwelling for 7-21±7 days in patients undergoing radical prostatectomy<br><br>**Observational:**<br>• Proportion of patients (%) who prematurely discontinued the study for any reason<br>• Measure and compare the biofilm formation on the surface of NO-impregnated versus non-impregnated Foley catheters after indwelling for 7-21±7 days<br>• Measure and compare bacteriuria (by urine culture) for NO-impregnated versus non-impregnated Foley catheters prior to insertion, on catheter insertion day, every day during hospitalization, on catheter removal day, and 30-45 days after catheterization<br>• Compare occurrences of clinically significant urinary tract infections (UTIs) following catheterization with NO-impregnated and non-impregnated Foley catheters<br>• Record different species of bacteria contaminations

<table>
<tr><th>Protocol number</th><th>ENOX_PHASE_I_1.1</th></tr>
<tr><td>Study End Points:</td><td><u>Primary End point(s):</u>

Safety-
• Determine AE's or SAE's associated with NO-impregnated catheters
• Proportion of patients (%) who prematurely discontinued the study due to AE's or SAE's

Tolerability-
• Proportion of patients (%) who prematurely discontinued the study due to AEs or SAEs associated with NO-impregnated catheter

<u>Observational</u>
• Proportion of patients (%) who prematurely discontinued the study for any reason
• Measure and compare the biofilm formation on the surface of NO-impregnated versus non-impregnated Foley catheters after indwelling for 7-21±7 days
• Measure and compare bacteriuria (by urine culture) for NO-impregnated versus non-impregnated Foley catheters prior to insertion, on catheter insertion day, every day during hospitalization, on catheter removal day, and 30-45 days after catheterization
• Compare occurrences of clinically significant UTIs following catheterization with NO-impregnated and non-impregnated Foley catheters
• Record different species of bacteria contaminations</td></tr>
</table>

<table>
<tr><th>Protocol number</th><th>ENOX_PHASE_I_1.1</th></tr>
<tr><td>Study Design:</td><td>Total of 12 patients undergoing radical prostatectomy will be enrolled into the study.
Prospective, Open label Phase I case series study: where 6 patients will be allocated to receive NO-impregnated Foley catheters (treatment) followed by 6 patients allocated to receive non-NO-impregnated Foley catheters (control).

1. <u>Screening:</u>
Candidate patients for radical prostatectomy will be screened for study eligibility by the study investigators. Eligible patients will be contacted and will be invited to participate in the study. The investigator will explain all aspects of the trial to the patient and will provide the patient with an informed consent form for review and approval.
Once the patient submitted a signed informed consent form, the inclusion and exclusion criteria will be verified.
Patients who meet all inclusion criteria and none of the exclusion criteria will be enrolled into the study.

2. <u>Treatment (7-21±7 days):</u>
Patients will be allocated to receive NO-impregnated Foley catheters or non-NO-impregnated Foley catheters for 7-21±7 days in addition to standard treatment that will be given in the hospital's Urology department and in the outpatient clinic.

3. <u>Telephone call follow-up visit (21±3 days)</u>
The patients will be contact by telephone call for a follow-up questionnaire.

4. <u>Follow up (30-45 days):</u>
The patients will be asked to attend the outpatient clinic 30-45 days after surgery in order to evaluate parameters subjected to the study.</td></tr>
<tr><td>Study Treatment Administration:</td><td>NO-impregnated Foley catheters and non-NO-impregnated Foley catheters will be supplied by ENOX Ltd.</td></tr>
<tr><td>Number of Patients:</td><td>Total of 12 patients
The trial will be open labeled where 6 patients will be allocated to receive NO-impregnated Foley catheters (treatment) followed by 6 patients allocated to receive non-NO-impregnated Foley catheters (control).
The planned sample size of 12 patients was considered adequate by the sponsor and investigators for this study. The study is not expected to show statistical significance or statistical power, only to demonstrate a safety profile.</td></tr>
</table>

**Protocol number**	**ENOX_PHASE_I_1.1**
**Inclusion/Exclusion Criteria:**	**<u>Inclusion Criteria:</u>**<br>1. Patients before radical prostatectomy at the Department of Urology in Beilinson Medical Center, which will be catheterized for 7-21±7 days.<br>2. Age:≥ 18 years.<br>3. Patients with a life expectancy of more than 12 months.<br>4. The investigator has completed a medical history and a physical examination to assure that the patients meets all study enrollment criteria.<br>5. The patient is willing and able to read, understand and sign the study specific informed consent form.<br><br>**<u>Exclusion Criteria:</u>**<br>1. A urinary culture demonstrating UTI before surgery.<br>2. A patient with an indwelling urinary catheter prior to surgery.<br>3. Expected life expectancy of less than 12 months.<br>4. Concurrent illness, disability or geographical residence that would hamper study participation.<br>5. Patients with underlying diseases such as heart disease, lung disease, skin disease or infection involving the penis, scrotum and groin, immunocompromised patients (transplant recipients, HIV carriers) or any other disease or condition that according to the physician opinion will influence the study results.<br>6. Patients with known urethral stricture.<br>7. Patients with recurrent UTIs.<br>8. Current participation in another clinical investigation of a medical device or a drug or has participated in such a study within 30 days prior to study enrollment.
**Route and Dosage Form:**	The patients will receive their standard treatment combined with the NO-impregnated Foley catheters or non-NO-impregnated Foley catheters for 7-21±7 days

Protocol number	ENOX_PHASE_I_1.1
**Statistical Considerations:**	All measured variables and derived parameters will be listed individually and, if appropriate, tabulated by descriptive statistics.<br>For categorical variables summary tables will be provided giving sample size, absolute and relative frequency and 95% Confidence Interval (CI) for proportions by study group.<br>For continuous variables summary tables will be provided giving sample size, arithmetic mean, standard deviation, coefficient of variation (if appropriate), median, minimum and maximum, percentiles and 95% CI for means of variables by study group.<br><br>**Statistical Methods:**<br>The study objective is to demonstrate a safety profile and tolerability. All adverse events will be coded according to coding dictionaries (MedDRA version 16.1 or higher) and presented in tables by System Organ Class (SOC) and Preferred Term (PT). Drug-related adverse events will be presented as well.<br>95% CI will be calculated for the proportion of patients who prematurely discontinued the study for any reason and due to any AE or SAE.<br>Chi-square test or Fisher's Exact test (as is appropriate) will be used for analyzing the difference in proportions between the study groups.<br>The Paired T-test or Signed rank test for two means (as is appropriate) will be applied for analyzing changes in continuous parameters within study group.<br>The two-sample T-test or Non-parametric Wilcoxon-Mann-Whitney Rank sum test for independent samples (as is appropriate) will be applied for analyzing differences in continuous parameters between the study groups.<br><br>All tests will be two-tailed, and a p-value of 5% or less will be considered statistically significant.<br>The data will be analyzed using the SAS ® version 9.1 (SAS Institute, Cary North Carolina).

2. LIST OF ABBREVIATIONS

Term	Description
AE	Adverse Event
CA	Competent Authorities
CRA	Clinical Research Associate
CAUTI	Catheter Associated Urinary Tract Infection
CBC	Complete Blood Count
CRO	Clinical Research Organization
CRF	Case Report Form
CFU	Colony Forming Unit
EC	Ethics Committee
EU	European Union
FDA	Food and Drug Administration
GCP	Good Clinical Practice
GMP	Good Manufacturing Practice
Hb	Hemoglobin
IB	Investigator's Brochure
ICH	International Conference on Harmonization
IRB	Institutional Review Board
IP	Investigational Product
IMP	Investigational Medicinal Product
MOH	Ministry Of Health
NO	Nitric Oxide
NOS	Nitric Oxide Synthase
NZW	New Zealand White
IDSA	Infectious Diseases Society of America
QA	Quality Assurance
QC	Quality Control
RNS	Reactive Nitrogen Species
SAE	Serious Adverse Event
SD	Standard Deviation
SOC	System Organ Class
SOP	Standard Operating Procedure
UTI	Urinary tract Infection

3. INTRODUCTION

3.1 Background

According to recent publications by the World Health Organization (WHO) [1], hundreds of millions of patients are affected by health-care associated infections worldwide each year, resulting in prolonged hospital stays, long-term disabilities, deaths, and financial losses for health systems. The most common hospital-acquired infection is urinary tract infection (UTI), which accounts for almost 40% of all nosocomial infections [2]. According to the Center of Disease Control (CDC), most hospital-acquired UTIs are associated with catheterization [3]. In fact, urinary catheter-related bacteriuria is the most common health care associated infection worldwide [4]. Studies have showed that *Escherichia coli* is the most common bacterial pathogen responsible for catheter-associated UTIs (CAUTIs) [5, 6].

Planktonic bacteria can adhere to surfaces on catheters and colonize, creating a persistent environment called a biofilm [7, 8]. These colonies consist of bacteria that are highly differentiated and resistant to standard antibiotics [9], augmenting the potential of these pathogens to cause infections in patients with indwelling catheters.

In recent years, catheters coated with various antiseptic materials have been investigated for their ability to prevent biofilm formation and bacteriuria [10-13]. Several manufactures have marketed either antimicrobial-impregnated or silver-coated catheters as representing a technology to reduce CAUTI risk. More specifically, Nitric oxide (NO), an endogenously produced gas molecule, which plays an important role in host defense against various pathogens, has also been investigated for its potential to prevent biofilm formation [14, 15].

Using a novel approach, Regev-shoshani et al., generated antiseptic barrier on urinary catheters by impregnating them with NO [16]. Upon immersion in urine, these catheters demonstrated slow release of NO over a 14-day period and were able to prevent bacterial colonization and biofilm formation on their luminal and exterior surfaces [14]. In addition, they were also shown to inhibit the growth of *Escherichia coli* within the surrounding media and eradicated bacterial concentrations of up to 10^4 CFU/ml, suggesting the high potential of NO to prevent CAUTIs.

Nitric Oxide

In 1987, endothelium derived relaxing factor was identified as nitric oxide (NO), a small lipophilic free radical gas molecule, that easily cross the plasma membrane into the cytosol [17, 18]. NO which is produced endogenously by various cell types including immune cells was shown to be involved in cell

signaling, vasodilatation of smooth muscle, neurotransmission, and regulation of wound healing. In addition, NO also plays a significant role in host defense against a large variety of pathogens [18-21].

NO is naturally synthesized in mammalian cells by an NADPH dependent nitric oxide synthase enzyme (NOS) which catalyzes oxidation of L-arginine to produce citrulline and NO [22]. Three isoforms of NOS are recognized in humans: two are constitutively expressed (endothelial NOS and neuronal NOS) and produce only nanomolar concentrations of NO. The third isoform, inducible NOS (iNOS), is expressed mainly by immune cells such as macrophages and neutrophils and is responsible for the high output of micromolar concentrations of NO following a variety of stimuli. In normal subjects, iNOS is up-regulated in macrophages and other immune cells following infection and/or stimulation by cytokines [21]. iNOS-induced elevation of NO together with other superoxide ions, produced by immune cells during inflammation results in the generation of several reactive nitrogen species (RNS) which are extremely toxic to bacteria. Indeed, during the last decade several mechanism by which NO and its RNSs elicits anti-bacterial and bactericidal activity were detected [21, 23-28]. These mechanisms were shown to affect bacteria in multiple compartments ranging from the outer membrane trough the cytoplasm and into the nucleolus. In addition, NO was also found to be involved in the prevention of biofilm formation and biofilm dispersal [14, 15, 29].

NO half-life (T1/2)

The half-life of NO, which corresponds to the time it takes to lose half of its pharmaceutical and physiological activity, is very short [17]. In the blood, NO disappears within seconds following binding with high avidity to hemoglobin (Hb). According to hakim et al., interaction between NO and Hb occurs in a 4:1 ratio, thus NO is unlikely to reach the body circulation as a free radical and to act as a circulating humoral substance [30]. In plasma fluids, NO react with molecular oxygen to form nitrite or with superoxide to form peroxynitrite ($ONOO^-$), which further decompose to nitrite and nitrate. It is assumed that reaction of NO with oxygen in aqueous solutions at a concentration typical of those that exist is cells are relatively slow, with a half-life for NO of about one hour [31].

Technologies used in urinary catheters to prevent infections

The most researched technical innovation in catheter design over the past 10 years has been the introduction of antimicrobial coatings applied to catheter surfaces or impregnated into the catheter material aimed to prevent bacterial attachment and biofilm formation [10-13]. Several manufactures have marketed

either antimicrobial-impregnated or silver-coated catheters as representing a technology to reduce CAUTI risk.

Silver has long been recognized as an antimicrobial agent with demonstrated activity against uropathogens through multiple mechanisms of action such as inactivation of vital enzymes by reacting with thiol groups, enhancing pyrimidine dimerization by photodynamic reaction, and causing cell wall changes induced by electron dense granules [13, 32]. A potentially more direct method of inhibiting CAUTI is to coat or impregnate catheters with antimicrobials active against expected uropathogens. An antimicrobial agent used for catheter impregnation is nitrofurazone, a topical nitrofuran related to nitrofurantoin, which has a spectrum of activity against many potential uropathogens, which involves interference with multiple bacterial intracellular targets including ribosomes, DNA and cell wall.

Study results have shown that silver impregnation had little effect on bacterial adherence, while nitrofurazone impregnation had a significant effect, yet only for the first 5 days following catheterization [33]. The results in the study do not support a role for silver urinary catheters to prevent CAUTIs by decreasing bacterial adherence. The results of this study and others lead to the conclusion that a solution of using a device that will be able to prevent UTIs for long time and without using antibacterial drugs has great potential for the health sector.

In a study funded by ENOX, Regev-Shoshani et al., showed that NO-impregnated catheters exhibited superior performance compared to silver-coated catheters, and similar anti-infective properties compared to antibiotic-coated catheters, suggesting that NO-impregnated catheters can act better to prevent bacteriuria than silver alloy–coated catheter and have a better antiseptic effect [16].

The use of NO in prevention of biofilm formation

The use of indwelling catheters is considered a major risk for UTI. A central part of the pathogenesis of CAUTIs involves the adherence and colonization of planktonic bacteria on catheters surface, creating a persistent environment called a biofilm. The nature of biofilm structure together with the physiological attributes of biofilm organisms confers an inherent resistance to various antimicrobial agents such as antibiotics, disinfectants or germicides. The mechanisms responsible for resistance include delayed penetration of antimicrobial agents through the biofilm matrix, altered growth rate of biofilm organisms, and other physiological changes due to the biofilm mode of growth [34].

In Vitro Studies

In recent years, catheters coated with various antiseptic materials have been investigated for their ability to prevent biofilm formation on catheters, and bacteriuria [10-13]. More specifically, several techniques which involve the release of NO were developed and studied for their potential to prevent biofilm formation on catheters. For example, in an *in vitro* model of a catheterized urinary bladder, Carlsson et al. showed that a combination of nitrite and ascorbic acid, which releases low concentration of NO (maximum of 10ppm), effectively killed a clinical isolate of *Escherichia coli*, and a reference strain after 24 hour exposure [15].

Using a novel approach, in a study funded by ENOX, Regev-shoshani et al. [16] generated antiseptic barrier on urinary catheters by impregnating them with NO. These NO-impregnated catheters were then immersed in urine to simulate a clinically relevant static environment. According to results, slow release of NO from NO-impregnated catheters during a 14 days period prevent bacterial colonization and biofilm formation of *Escherichia coli* on their luminal and exterior surfaces. In addition, NO was able to eradicate up to 10^4 CFU/ml of bacteria within the surrounding media [14]. In another study, Regev-Shoshani et al. showed that NO-impregnated and antibiotic Nitrofurazone (NF)–coated catheters were equivalent in their antimicrobial activity and eradicated all bacteria in planktonic and biofilm states [16].

The mechanism by which NO prevents biofilm formation beyond simple eradication of the biofilm forming bacteria needs to be further clarified, yet it is speculated that NO prevents the attachment of bacteria and inhibition of biofilm formation in a mechanism which involves reduction and modification of proteins that mediate cell-substrate and cell-cell interactions [35-37].

In light of the results obtained by Regev-Shoshani et al., which showed that NO-impregnated catheters exhibited superior performance compared to silver-coated catheters, and similar anti-infective properties, compared to antibiotic-coated catheters, we propose that NO-impregnated catheters can act better to prevent bacteriuria than silver alloy–coated catheter and have a better antiseptic effect. These data highlights the great potential of NO in the prevention of biofilm formation, an attribute that might ease the burden of CAUTIs on health systems. Finally, the high efficiency of NO-impregnated catheters in preventing planktonic growth and biofilm formation is consistent and supportive of the Infectious Diseases Society of America (IDSA) guidelines.

NO-impregnated catheters Animal safety trials

In February, 2014, ENOX conducted safety trials for the NO-impregnated catheters were in NZW rabbits. The experimental design included two equally-

sized groups (n=5, each group): one group was subjected to urethral catheterization with NO-impregnated indwelling urinary catheter, while the control group was subjected to urethral catheterization with non-impregnated indwelling urinary catheter. Safety-related parameters were evaluated following an exposure period of 7 successive days to the indwelling urinary catheter.

Study results:

- Mortality events did not occur in both experimental groups prior to the scheduled termination time point
- No treatment-related adverse reactions were observed among all experimental animals following the urethral catheterization and throughout the entire 7-day observation period
- No marked differences were observed in the mean group body weight values between both experimental groups, determined prior to the urethral catheterization and at the end of the 7-day observation period
- There were no statistically significant differences in mean group hematology and biochemistry values, body weights and rectal temperatures among the test device-treated group *vs.* the respective values determined in the control device animals
- Local urethra mucosal irritation was found similarly in both groups

According to electron microscopy images, obtained at the end of the 7-day catheterization period, it seems that sections from NO-impregnated catheters exhibited reduced biofilm formation on the exterior and luminal surfaces, compared to control group. Although the concentration of the biofilm-forming bacteria was not examined in this study, it is likely that NO-impregnated catheters may have a beneficial effect on the ability of bacteria to form biofilm on catheter surface.

3.2 Study Hypothesis

NO-impregnated Foley catheters indwelled for 7-21±7 days are safe and tolerable.

3.3 Summary

This study proposes to use NO-impregnated catheters takes into consideration previous knowledge of:

(a) The known infection prevention properties of NO-impregnated catheters (ENOX innovation method).

(b) Nitric oxide activity as vasodilator.

(c) NO's very short half-life within the blood system, which minimize the time of active NO presence in the blood and its potential hazard to distal organs.

(d) NO-impregnated catheters have a safe profile in a rabbit model.

(e) Long term use of NO in a gas formation which has been approved by FDA to treat premature infants through mechanical respiratory rout.

NO-impregnated catheter method was developed by ENOX to prevent nosocomial infection. Bacterial colonization rates in catheterized patients are high. Moreover, bacterial density and biofilm formation in the catheter is high and consistent for days/weeks (but tend to increase over time). NO-impregnated catheters reduces bacterial density and can prevent biofilm formation [16], and were found to be safe in animal trials.

4. STUDY OBJECTIVES

4.1 Primary Objective

Safety and Tolerability:

- Assess the safety of NO-impregnated Foley catheters indwelling for 7-21±7 days in patients undergoing radical prostatectomy
- Assess the tolerability of NO-impregnated Foley catheters indwelling for 7-21±7 days in patients undergoing radical prostatectomy

4.2 Observational

- Proportion of patients (%) who prematurely discontinued the study for any reason
- Measure and compare the biofilm formation on the surface of NO-impregnated versus non-impregnated Foley catheters after indwelling for 7-21±7 days
- Measure bacteriuria (by urine culture) for NO-impregnated versus non-impregnated Foley catheters prior to insertion, on catheter insertion day, every day during hospitalization, on catheter removal day, and 30-45 days after catheterization
- Compare occurrences of clinically significant UTIs following catheterization with NO-impregnated and non-impregnated Foley catheters
- Record different species of bacterial contaminations

5. STUDY END POINTS

5.1 Primary End Point(s)

Safety:

- Determine AEs and SAEs associated with NO-impregnated catheters
- Proportion of patients (%) who prematurely discontinued the study due to AEs or SAEs

Tolerability:

- Proportion of patients (%) who prematurely discontinued the study due to AEs or SAEs associated with NO-impregnated catheter

5.2 Observational

- Proportion of patients (%) who prematurely discontinued the study for any reason
- Measure and compare the biofilm formation on the surface of NO-impregnated versus non-impregnated Foley catheters after indwelling for 7-21±7 days
- Measure and compare bacteriuria (by urine culture) for NO-impregnated versus non-impregnated Foley catheters prior to insertion, on catheter insertion day, every day during hospitalization, on catheter removal day, and 30-45 days after catheterization
- Compare occurrences of clinically significant UTIs following catheterization with NO-impregnated and non-impregnated Foley catheters
- Record different species of bacterial contaminations

5.3 Safety and Tolerability

All measurements will be done continuously to avoid exceeding allowed range of the up mentioned parameters.

5.3.1 Vital Sign Measurements

Full physicals examination will be performed by a study physician on each visit and every day during hospitalization (visit 2). Abbreviated physical examination (vital signs) by a study coordinator or by the medical staff will be done on each visit and during hospitalization 3 times per day.

Cardiovascular status will be determined by monitoring heart rate and blood pressure. Values will be recorded every 8 hours (3 times per day).

* Vital signs measurement includes the followings parameters: body temperature, blood pressure and heart rate.

5.3.2 Laboratory Tests: Blood & Urine

Blood:

- Complete Blood Cells Count (CBC).
- Blood biochemistry (electrolytes, renal and liver function tests).
- Coagulation tests (PT, aPTT, INR).

Urine:

- Urine sterile specimen for culture will be obtained prior to insertion, on catheter insertion day, every day during hospitalization, on catheter removal day, and 30-45 days after catheterization
- Urine for biochemical tests including nitrate concentration

6. STUDY DESIGN

6.1 Overview and Plan

This is a Prospective, Phase I, Open-label Case Series, Single-Center study.

A total of 12 patients, over the age of 18, undergoing radical prostatectomy will be enrolled. Screening data will be reviewed to determine patients' eligibility and informed consents will be obtained.

Patients who meet all inclusion criteria and none of the exclusion criteria will be enrolled.

6 patients will be allocated to receive NO-impregnated Foley catheters (treatment) followed by 6 patients allocated to receive non-NO-impregnated Foley catheters (control).

A patient will receive catheter up to 21±7 days of study treatment. A patient is considered as completed the study "per protocol", once study treatment was completed: 30-45 days after catheterization.

Treatment:

Patients enrolled to the study will be administered NO-impregnated or non-NO-impregnated indwelling catheter for 7-21±7 days in addition to standard treatment given in the hospitals urology department and in the outpatient clinic.

6.2 Study design and Scheduled Visits (Visit 1 – Visit 5)

	Screening and Enrollment (day 0)	Day of Operation and Catheterization (day 1)	Hospitalization (day 2 - discharge)	Catheter removal (day 7-21±7 days)	follow up Telephone call (day 21±3 days)	Follow up visit (day 30 –45)
Visits	Visit 1	Visit 2		Visit 3	Visit 4	Visit 5
Screening, signing informed consent and Enrollment	+					
Physical exam	+	+	+*	+		+
Catheter insertion		+				
Urine microbiological test (CFUs)	+	+	+*	+		+
Lab tests (CBC, biochemistry, bleeding)	+	+	+*	+		+
Vital signs**	+	+	+*	+		+
Catheter removal				+***		
Post void residual urine volume				+		
Questioner for demographics data and medical history	+					
Telephone call					+	

* Every day during hospitalization. At physical examination put emphasis on penis tissues such as the glans, shaft and prepuce.

** Vital signs: heart rate, blood pressure, and temperature. During hospitalization, these will be done every 8 hours.

*** After catheter removal catheter specimens will be processed and evaluated for bacterial biofilm (see Appendix 1).

7. STUDY POPULATION

7.1 Number of Patients

A total of 12 patients, over the age of 18 undergoing radical prostatectomy will be enrolled.

The study is not expected to show statistical significance or statistical power, but to demonstrate a safety and tolerability profile.

6 patients will be catheterized with NO-impregnated Foley catheters followed by 6 patients catheterized with non-NO-impregnated Foley catheters.

7.2 Inclusion Criteria

- Patients before radical prostatectomy at the urology department in Beilinson Medical Center, which will be catheterized for 7-21±7 days
- Age: ≥18 years
- Expected life expectancy of more than 12 months
- The investigator has completed a medical history and a physical examination to assure that the patient meets all study enrollment criteria
- The patient is willing and able to read, understand and sign the study informed consent form

7.3 Exclusion Criteria:

- A urinary culture demonstrating UTI before surgery
- A patient with an indwelling urinary catheter prior to surgery
- Life expectancy of less than 12 months
- Concurrent illness, disability or geographical residence that would hamper study participation
- Patients with underlying diseases such as heart disease, lung disease, skin disease or infection involving the penis, scrotum and groin, immunocompromised patients (transplant recipients, HIV carriers)
- Patients with known ureteral stricture
- Patients with recurrent UTIs
- Current participation in another clinical investigation of a medical device or a drug or has participated in such a study within 30 days prior to study enrollment

7.4 End of Treatment Assessments (after completing 7-21±7 days of study catheterization)

End of study assessment include:

- AEs and SAEs
- Bacterial detection on catheters and culture from catheters and urine
- Vital signs measurements

7.5 Early Treatment Discontinuation

End of treatment assessments should be completed for all patients who prematurely discontinue from the study. This includes patients who were screened even though they were not exposed to the NO-impregnated catheter.

Every attempt should be made to follow the early discontinued patients according to the regular scheduled visits as per protocol. The patients who prematurely discontinue the study should be followed and treated by the investigator in a customary manner. Patients who decide not to continue with any further visits should complete the end of treatment assessment discontinuation.

7.5.1 Criteria for Treatment Discontinuation

- AEs that are suspected to be related to NO-impregnated catheters, according to physician discretion

- Blood pressure and heart rate above or below the 20% of the normal ranges for the patient baseline

A patient whose treatment was discontinued for any reason should complete all study assessment including the follow up visits.

Concomitant medication (i.e. antibiotics) given during the study will not be a reason for treatment early discontinuation.

7.5.2 Criteria for Early Study Withdrawal

A patient may withdraw or be withdrawn from the study for the following reasons:

1) Patient withdrew consent.
2) Protocol violation/ non-compliance.
3) Loss to follow-up/failure to return.
4) Patient with difficulty in insertion of catheter during surgery.
5) In cases which require catheter replacement.
6) Serious adverse event

7.5.3 Criteria for Study Stop

In case of death, the study will be stopped for medical re-evaluation.

7.5.4 Replacement of Withdrawn Patients

Up to 12 patients will be recruited, in order to have a sample size of at least 6 patients catheterized with NO-impregnated catheters and 6 patients catheterized with non-NO-impregnated catheters, who completed the study (see protocol).

8. STUDY CONDUCT DETAILED STUDY PLAN

8.1 Screening & Enrolment procedures

8.1.1 Screening and Enrolment – Visit 1 (day 0)

- Check patient eligibility (Inclusion/exclusion criteria)
  Enroll only patients who meet all inclusion criteria and none of the exclusion criteria
- Review study with the patient and obtain written informed consent
- Record demographics data and medical history
- Record concomitant medications
- Perform a full physical examination
- Perform urine test (microbiology and biochemistry), blood test for CBC, biochemistry, and coagulation: Prothrombin Time (PT), activated Partial Thromboplastin Time (aPTT), and International Normalized ratio (INR)
- Perform and record vital signs: temperature, blood pressure and heart rate
- Document any laboratory test done for medical reasons during the preceding month (blood tests, urinalysis etc.)

8.2 Randomization

N/A

8.3 Study Treatment

8.3.1 Operation and Catheterization – Visit 2 (day 1)

- Perform a full physical examination
- Perform and record vital signs: temperature, blood pressure and heart rate
- Catheter insertion
- Perform urine test (microbiology and biochemistry), blood test for CBC, biochemistry, and coagulation: Prothrombin Time (PT), activated Partial Thromboplastin Time (aPTT), and International Normalized ratio (INR)

8.3.2 Hospitalization – Visit 2 continuation (day 2 - discharge day)

- Perform a full physical examination
- Perform and record vital signs: temperature, blood pressure and heart rate
- Perform urine test (microbiology and biochemistry) , blood test for CBC, biochemistry, and coagulation: Prothrombin Time (PT), activated Partial Thromboplastin Time (aPTT), and International Normalized ratio (INR)

8.3.3 Catheter Removal – Visit 3 (day 7-21±7)

- Remove Catheter**
- Perform a full physical examination
- Perform and record vital signs: temperature, blood pressure and heart rate
- Perform urine test (microbiology and biochemistry) , blood test for CBC, biochemistry, and coagulation: Prothrombin Time (PT), activated Partial Thromboplastin Time (aPTT), and International Normalized ratio (INR)
- Measure post-void residual urine volume

** After catheter removal catheter specimens will be processed according to protocol (see appendix 1).

8.4 Follow-up Visits

8.4.1 Telephone call – Visit 4 (day 21±3)

- Perform a telephone call for follow-up and record any new medical conditions, AEs, or SAEs

8.4.2 Follow-up Visit - Visit 5 (day 30- 45)

- Perform a full physical examination
- Perform and record vital signs: temperature, blood pressure and heart rate
- Perform urine test (microbiology and biochemistry) , blood test for CBC, biochemistry, and coagulation: Prothrombin Time (PT), activated Partial Thromboplastin Time (aPTT), and International Normalized ratio (INR)

8.5 Unscheduled Visits

An unscheduled visit may be performed at any time during the study at the patient's request or as deemed necessary by the investigator. The date and reason for the unscheduled visit will be recorded. The following procedures and evaluations may be completed as deemed necessary by the investigator:

- Perform a full physical examination
- Perform and record vital signs: temperature, blood pressure and heart rate
- Perform urine test (microbiology and biochemistry) , blood test for CBC, biochemistry, and coagulation: Prothrombin Time (PT), activated Partial Thromboplastin Time (aPTT), and International Normalized ratio (INR)

8.6 Termination/Study Early Discontinuation

Treatment/study can be either temporarily or permanently discontinued. Criteria for discontinuation are specified in section 7.5

The reason for discontinuation of study will be documented in the source documents and captured on the CRF. In the event that the catheter is removed prematurely, end of treatment assessments (section 7.4) should be completed as close to the last date as possible.

If a patient is withdrawn because of an AE, the appropriate "Withdrawal Section" of the CRFs should be fully completed in addition to the AE module. ENOX BIOPHARMA should be informed of all patients who are withdrawn for this reason.

Documented attempts will be made to follow a patient who prematurely discontinues the study.

8.7 Emergency Code Breaking

N/A

9. DESCRIPTION OF INVESTIGATIONAL MEDICINAL PRODUCTS/STUDY

9.1 Foley Catheter Embedded with Nitric Oxide

The catheters are manufactured and bought from Biometrix Ltd, an Israeli supplier. These catheters are approved for use in the Israeli health system (see appendix 2). The catheters are already sterile and ready to use.

For the study we will impregnate 18Fr catheters. This diameter was chosen follow consultation with the principal investigator of the study.

9.2 Impregnating Device

To impregnate the NO gas into the catheter, a designated proprietary impregnating device is used. The impregnating device is a chamber that is connected to vacuumed pump and an NO gas container. The gas that is used is supplied by Maxima Ltd, Israel, which is responsible for its manufacturing. The gas, which is tagged and colored according to the israeli regulation, is stored at 20,000 ppm, concentration with 99.999% nitrogen purity - according to Good Manufacturing Practice (GMP) spirit and guidelines and ISO 9001.

9.3 Impregnating Process

Catheters (see appendix 3) wrapped in their original sterile package are placed inside the impregnating chamber. After the chamber is tightly secured the vacuum pump is connected to the vacuum outlet of the chamber. Vacuum pump is then turned on, and the vacuum outlet is opened until the pressure reaches negative 10 psi. When required pressure is achieved the vacuum outlet is closed and the vacuum pump is turned off. Then the gas cylinder, containing 20,000ppm is turned open, while the regulator is open and is connected to the chamber. The NO gas, stored in purple cylinder is let flow into the impregnating chamber through the opened gas inlet, until the pressure reaches back to the level of 0 psi. The gas supply is closed from the gas cylinder, while the regulator and the process valve on the chamber are closed. The catheters are impregnated by incubation with 20,000ppm NO for at least 4 hours. Once impregnating is completed the "purge" outlet is opened, and air flows from condensed air (green cylinder) cylinder. During this time the regulator is directed to provide a very low pressure of 1-2 psi, during purging of at least 2 hours. Then the lid is released and the chamber is emptied from the loaded device

9.4 Sterilization/Validation

During the entire impregnating process the catheters are kept in their original sterile packages. Therefore, at the end of the impregnating process the catheters are expected to remain intact and sterile.

In order to confirm that the catheters are not contaminated during the impregnating process, ENOX conducted a sterilization/validation test according to current GMP for medical devices. The tested catheters met all sterilization criteria and were found to be sterile (see appendix 4).

10. RISK ANALYSIS

In light of the low concentration expected to be released from the catheter, and in light of the short half-life of NO in the blood, we do not expect any severe and systemic side effects. However, low concentration of inhaled NO (5-40ppm) in some cases was found to be associated with decreased blood pressure, inhibition of platelet aggregation, and increased bleeding time [38-40]. In addition, NO may trigger local side effects such as erection, local skin irritation, and the formation of skin edema and erythema [41] . (See appendix 5 - table of possible adverse events related to NO-impregnated catheters).

To assess the risk associated with the use of NO-impregnated catheters, ENOX performed a pre-clinical safety study in NZW rabbits. No adverse effect and no safety concerns were raised in the conclusion of this study (see main content of the report in appendix 2 in the investigator brochure). ENOX chose to conduct the safety trial on rabbits and not on larger animals such as pigs for two main reasons: 1) the life habitat of pigs is unsterile by nature and may affect trial outcome, causing undesired manifestations that are not related to NO-impregnated catheters. 2) The anatomy of pigs' urethra is not ideal for catheterization over an extended period

Additional tests performed by ENOX include:

- Mechanical test, performed by degania silicone Ltd., Israel
- In vitro cytotoxicity test performed by Harlan Biotech, Israel (STUDY NO ENI/001/CTX)
- In vivo intradermal reactivity tests performed by Harlan Biotech, Israel (STUDY NO ENI/002/IRT)
- In vivo sensitization test performed by Phycher Bio Developpment, France (Harlan Project NO 41400562)
- In vivo safety trial for the NO-impregnated catheters performed by Harlan Biotech, Israel (STUDY NO ENI/003/EM)

ENOXs NO-impregnated catheter meet with all required criteria and was classified as non-cytotoxic, non-irritating and non-sensitizing device (see full reports in IB).

During the trial catheterized patients will be monitored daily (see protocol, section 6.1) through physical examination and vital signs measurements. In addition, blood tests will also be conducted in order to exclude any significant change in platelet counts, and biochemistry values (see appendix 5 - table of possible adverse events related to NO-impregnated catheters).

11. ASSESSMENT METHODS

11.1 Observational

- Proportion of patients (%) who prematurely discontinued the study for any reason
- Measure and compare the biofilm formation on the surface of NO-impregnated versus non-impregnated Foley catheters after indwelling for 7-21±7 days
- Measure and compare bacteriuria (by urine culture) for NO-impregnated versus non-impregnated Foley catheters prior to insertion, on catheter insertion day, every day during hospitalization, on catheter removal day, and 30-45 days after catheterization
- Compare occurrences of clinically significant UTIs following catheterization with NO-impregnated and non-impregnated Foley catheters.
- Record Different species of bacteria contaminations

11.2 Medical/Clinical Assessment

Initial demography/medical history form (complete questionnaire including AE and SAE will be provided).

Physical Examination:

A physical examination will be performed by a physician (either the principal investigator or a sub-investigator) on each visit. Body systems to be examined grossly include: General, Skin, Lymph Nodes, HEENT, Respiratory, Cardiovascular, Gastrointestinal, Neurologic and Muscoskeletal, and the Penis, Scrotum and Groin. New abnormal findings must be documented and should be followed by an investigator at the next scheduled visit.

Vital Signs:

Heart rate, blood pressure, and temperature will be performed and recorded.

Adverse Events:

Information regarding occurrence of AEs will be captured throughout the study and until the patients follow-up is complete.

Event duration (start and stop dates and times), severity, outcome, treatment and relation to study medication (causality) and if the event is regarded as SAE, will be recorded in the CRF.

AEs will be followed until day 30-45 of the study.

Concomitant Medications:

Concomitant medication given during the study will not lead to study treatment discontinuation.

All concomitant medication and concurrent therapies will be documented throughout the study until day 30 of the study.

The following information will be recorded: Dose, route, unit frequency of administration, and indication (if deemed relevant) for administration of medication. All concomitant procedures will be documented throughout the study until day 30 of the study, the reason for the procedure will be considered as AE/SAE, unless it was scheduled prior to study start.

11.3 Clinical Laboratory Assessments/Evaluations

11.3.1 Blood Tests

Blood tests as detailed in section 5.3.2 to this protocol will be recorded in the CRF and will be used for the study analysis.

Any other blood tests results (that were done for clinical reasons and not required for study) collected during the patient's treatment or study follow up will be recorded in the CRF and will be used for observational analysis.

11.3.2 Bacteriological Tests

Detection of bacteria in urine:

Urine tests for relevant bacteria will be performed according to the European Urinalysis Guidelines published by the European Confederation of Laboratory Medicine (ECLM) [42], in consultation with the microbiologist on site.

Detection of bacteria on catheter

Within removal of the catheter (1 hour maximum), cut the catheter into small 1-cm sections. 3 x 1-cm sections from each catheter will be analyzed per tests for biofilm formation as followed:

Exterior catheter surface:

Within removal of catheter (1 hour maximum), cut the catheter is to small sections of 1cm each. Then the catheter section is washed three times using 3 ml of sterile saline (0.9% wt/vol NaCl). After wash, the catheter section is

aseptically transferred to an LB agar plate, and rolled once on the plate. The plate is then incubated at 37°C for 24 hours.

Quantification of biofilm and Biofilm-forming bacteria:

Quantitative measurement of bacterial biofilm and biofilm-forming bacteria will be determined by two methods: absorbance at 595nm using a spectrophotometer, and CFU measurement following sonication.

Spectroscopy:

A 1-cm section of the catheter is washed 3 times with saline and added to 1.5 ml of crystal violet dye (serves as an indicator of biofilm formation) previously diluted in water (1% wt/vol). After 15 minutes incubation the catheter section is washed three times with water and transferred to 2 ml of 95% ethanol to relinquish crystal violet bound to the surface of the catheter. Absorbance of each ethanol sample is read at 595 nm using a spectrophotometer.

CFU measurements:

A 1-cm section of the catheter is washed 3 times with saline. Transfer each section to 2 ml of saline 0.9% and sonicate for 20 seconds (30KHz). CFU’s are determined by plating each sample on blood agar after serial dilution with saline and incubation at 37°C overnight.

11.4 Safety Parameters

11.4.1 Adverse Events

Adverse events will be recorded from the date of patients signed informed consent form and throughout the study, including the follow-up period. They should be reviewed and updated at each subsequent visit and during any phone contact with the patient.

11.4.2 Potential Adverse Events Related to NO-impregnated Catheters

- Bleeding (i.e hemoptysis)
- Decrease in blood pressure
- Erection
- Local irritation

(See appendix 5 - table of possible adverse events related to NO-impregnated catheters).

11.4.3 Safety Laboratory Evaluations

Bleeding test such as prothrombine time (PT), international normalized ratio (INR), activated partial thromboplastin time (aPTT) will be performed (see section 8)

12. SAFETY

Any untoward event that occurs, whether or not the investigator considers the event to be related to the use of the NO-impregnated catheters, and any new condition or worsening of a pre-existing condition will be considered an AE. Stable chronic conditions that are present prior to study entry and do not worsen during the study will not be considered AEs.

An abnormal result of diagnostic procedures, including abnormal laboratory findings, will be considered an AE if it:

- Results in patient's withdrawal by the investigator
- Is associated with a SAE
- Is associated with clinical signs or symptoms
- Is considered by the physician to be of clinical significance

The intensity or severity of the AE will be characterized as:

Mild: AE which is easily tolerated
Moderate: AE sufficiently discomforting to interfere with daily activity
Severe: AE which prevents normal daily activities.

The causality of the AE will be assessed as:

Unrelated: when the AE is clearly and incontrovertibly due to extraneous causes (disease, environment, etc.).

Unlikely related: if the AE meets at least two of the following criteria: 1) it does not follow a reasonable temporal sequence from catheterization; 2) it could readily have been produced by the patient’s clinical state, environmental or toxic factors, or other modes of therapy administered to the patient; 3) it does not follow a known pattern of response NO.

Possibly related: if the AE meets at least two of the following criteria: 1) it follows a reasonable temporal sequence from catheterization; 2) a causal relationship to the experimental treatment cannot necessarily be reasonably excluded and an alternative explanation (e.g., concomitant drug or concomitant disease) cannot be reasonably suggested as causing the SAE; 3) it follows a known pattern of response to NO.

Probably related: if the AE meets at least three of the following criteria: 1) it follows a reasonable temporal sequence from catheterization; 2) it cannot be reasonably explained by the known characteristics of the patient’s clinical state, environmental or toxic factors, or other modes of therapy administered to the patient; 3) it disappears or decreases on catheter removal, and it follows a known pattern of response to NO.

The date of onset, a description of the AE, severity, seriousness, action taken, relationship to the NO-impregnated catheter, outcome of the event, and date of resolution will be recorded. It should be documented if considered an SAE.

An SAE is defined as an AE that

- Results in death
- Is life-threatening
- Requires re-hospitalization or prolongs existing inpatient hospitalization
- Results in persistent or significant disability or incapacity
- Is an significant medical event which requires medical intervention to prevent any of the above outcomes

Significant medical events are those that may not be immediately life-threatening, but may jeopardize the patient and may require intervention to prevent one of the other serious outcomes listed above. Occurrences resulting in an AE will normally be considered serious by this criterion.

Inpatient hospitalization, or prolongation of existing hospitalization, mean that hospital inpatient admission and/or prolongation of hospital stay were required for treatment of AE, or that they occurred as a consequence of the event. It does not refer to pre-planned elective hospital admissions for treatment of a pre-existing condition that has not significantly worsened, or to any diagnostic procedure.

Any new SAE that occurs after the study period and is considered to be related (possibly/probably) to the study participation should be recorded and reported immediately, as requested by the Israeli Ministry of Health (MOH).

In order to satisfy regulatory requirements, any SAE, whether deemed IP-related or not, must be reported to the sponsor and to the study monitor as soon as possible after the investigator or coordinator has become aware of its occurrence. The SAE form completion and reporting must not be delayed even if all of the information is not available at the time of the initial contact.

The SAE should be submitted to the sponsor within 24 hours of becoming aware of the event. Additional information (follow-up) about any SAE unavailable at the initial reporting should be forwarded by the site to the sponsor within 24 hours of the information becoming available.

The SAE report (initial and/or follow-up) should be sent via e-mail to:

dudi@bgu.ac.il

AND faxed to fax number: +972-4-6533471

The e-mail or the fax confirmation should be filed in the Investigator Site File.

The SAE will be reported to the regulatory authorities (CA) and EC/IRBs, according to Israeli Ministry of Health regulations (MoH form 13 or the SAE report form can be used, as requested by the IRB).

The following information should be provided to accurately and completely record the event:

1) Investigator name
2) Patient number
3) Patient initials
4) Patient demographics
5) Clinical event
    - Description of the event
    - Date of onset
    - Severity of the event
    - Treatment
    - Relationship to study catheter (causality)
    - Actions taken regarding study catheter
6) If the AE results in Death
    - Cause of death (whether or not the death is related to study catheter)
    - Autopsy findings (if available)
7) Medical history CRF (copy)
8) Concomitant medication case report (copy of relevant CRF page)
9) Any relevant reports (laboratory, discharge, x-Ray, etc.)

This information should be sent to the sponsor.

The procedures for notification of suspected serious unexpected adverse reactions (SUSARs) shall be carried out in accordance with the Israeli MOH. SAEs should be reported by the ENOX BIOPHARMA to EC/IRB according to local requirements.

Patients who have had an SAE during the treatment period must be followed clinically until all parameters (including laboratory) have either returned to normal or have stabilized or are otherwise explained.

Any newly emergent SAEs, after treatment is discontinued or the patient has completed the study and is considered to be related to the investigational medicinal product (IMP) or study participation, should be recorded and reported immediately. The post-study period for the purpose of SAE reporting is routinely up to 30 days following last study visit or until SAE is resolved or stabilized.

13. STATISTICAL METHODOLOGY

General:

All measured variables and derived parameters will be listed individually and, if appropriate, tabulated by descriptive statistics.

For categorical variables summary tables will be provided giving sample size, absolute and relative frequency, and 95% CI for proportions, by study group.

For continuous variables summary tables will be provided giving sample size, arithmetic mean, standard deviation, coefficient of variation (if appropriate), median, minimum and maximum, percentiles, and 95% CI for means of variables, by study group.

Safety and Tolerability:

The study objective is to demonstrate a safety and tolerability profile.
Adverse events will be coded according to coding dictionaries (MedDRA version 16.1 or higher) and presented in tables by System Organ Class (SOC) and Preferred Term (PT). Additional tables will be generated for adverse events associated with NO-impregnated catheters.

95% CI will be calculated for the proportion of patients who prematurely discontinued the study for any reason and due to any AE or SAE.

Chi-square test or Fisher’s Exact test (as is appropriate) will be used for analyzing the difference in proportions between the study groups. Laboratory results will be summarized in appropriate tables by time. Changes from baseline will be calculated and presented as well.

Observational Assessment

95% CI will be calculated for the proportion of patients with catheter-related urinary tract infections at any time point.

Chi-square test or Fisher’s Exact test (as appropriate) will be used for analyzing the difference in the above proportions between the study groups.

The Paired T-test or Signed Rank test for two means (paired observations; as is appropriate) will be applied for testing the statistical significance of the changes from baseline to any post-baseline measurement in biofilm formation on the surface and bacteriuria within study group.

The two-sample T-test or Non-parametric Wilcoxon-Mann-Whitney Rank sum test for independent samples (as appropriate) will be applied for testing the statistical significance of the differences in the above parameters between the study groups.

All tests will be two-tailed, and a *p-value* of 5% or less will be considered statistically significant.

The data will be analyzed using the SAS ® version 9.1 (SAS Institute, Cary North Carolina).

13.1 Sample Size Rationale

Total of 12 patients.

The planned sample size of 12 patients for the Phase I trial took under consideration the possibility of a 10% drop, as detailed in section 7.5. The planned sample size considered adequate by the sponsor and investigators for this study. The study is not expected to show statistical significance or statistical power, only demonstrate a safety profile.

13.2 Randomization

N/A

13.3 Patient Cohorts

Intent-to-Treat Cohort (ITT):

The ITT cohort includes all patients who receive at least one day of catheter treatment.

Completer Cohort:

The Completer cohort includes all patients in the ITT cohort who complete the study.

Per Protocol Cohort:

The Per-Protocol cohort includes all patients in the ITT cohort who complete the study (completed study treatment) in compliance with the protocol and have no major protocol violations.

For the ITT cohort, the Last Observation Carried Forward (LOCF) approach will be applied, when deemed appropriate, to account for missing data at or prior to study termination.

13.4 Comparability of Treatment Groups at Baseline

Primary End Points - as in section 5.1

Observational End Points - as in section 5.2

Safety and Tolerability Assessments

Adverse Events: The incidence and frequency of AEs will be presented by SOC and preferred terminology according to MedDRA dictionary. AEs will also be presented by SOC, High Level Term and preferred terminology.

Data will be tabulated by treatment group, age, maximal severity, maximal outcome, maximal action taken, and maximal relationship to the tested catheters.

SAEs and seriousness criteria will be listed and discussed on a case by case basis.

Laboratory Tests: The incidence of laboratory tests outside the normal range and the incidence of measurements of potential clinical significance will be presented by treatment group. Shift analysis of these counts from baseline will also be provided. Descriptive statistics as well as their changes from baseline will also be presented by study group.

Vital Signs: Incidence of measurements of potential clinical significance will be presented by study group. Shift analysis from baseline will be provided as well. Descriptive statistics of vital signs, as well as, their changes from baseline, will be presented by study group.

Tolerability Assessments

Tolerability analysis will be based on the number (%) of patients who failed to complete the study, the number (%) of patients who failed to complete the study due to AEs. Time to withdrawal will be presented by Kaplan-Meier curves. Significance testing of time to withdrawal will be done using Cox's proportional hazards model.

14. REGULATORY AND ETHICAL ISSUES

14.1 Compliance with Regulations Applicable to Clinical Trials

The study will be conducted according to the laws, regulations and administrative provisions relating to the implementation of good clinical practice in the conduct of clinical trials on medicinal products for human use, as applicable by national legislation (according to the Israeli Ministry of Health regulations) and EU Directives and US 21 CFR Part 11, 50, 54, 56, 312.

14.2 Informed Consent

The principles of Informed Consent, according to the Declaration of Helsinki 1964 and its updates up to 1996, ICH guidelines on GCP, 21 CFR part 50 of the FDA Regulations and/or EU Directives, will be followed. The Israeli Ministry of Health template of inform consent form will be used. A patient should not enter a clinical study until he have been properly informed, have been given time to contemplate participation, and have freely given consent by signing and dating the Institutional Review Board (IRB) approved informed consent form. This must be done prior to performing any study related procedures.

The proposed consent form and any other documents relevant to the consent process must be submitted to the IRB, together with the protocol, and must be approved prior to study start.

A copy of the fully signed and dated informed consent form and any other documents relevant to the consent process will be given to the patient and the original will be maintained at the site.

The informed consent process should include an explanation in the language the patient knows fluently. The consent form should be in a language the patient can read and understand. The process should be documented in the patient's file.

Any deviation from the consent process should be approved by the IRB as well as documented.

14.3 Institutional Review Board (IRB)

The study must have unconditional approval in writing, by an appropriate Review board (IRB). A copy of the letter of approval from the IRB, which contains specific identification of the documents approved, must be received by ENOX prior to site initiation.

Any amendments to the protocol or subsequent changes to the informed consent form as a result of changes to the protocol and/or investigator brochure that is approved by ENOX , must also be approved by the IRB and documentation of

this approval provided to ENOX. Records of the IRB review and approval of all documents pertaining to this study must be kept on file by the Investigator and are patient to the sponsor's audit and/or regulatory authority inspection, during or after completion of the study.

SAEs will be reported to the IRB by the investigator according to Israeli MOH regulations and the IRB requirements

Periodic status reports must be submitted to the IRB as required, as well as notification of completion of the study and a final report where applicable. A copy of all reports submitted to the IRB must be sent to the sponsor.

14.4 Protocol Amendments

Protocol amendments issued by the sponsor must be approved by the IRB and the Israeli Ministry of Health prior to implementation.

14.5 Declaration of the End of the Clinical Trial

A declaration of the end of the clinical trial will be made according to Israeli Ministry of Health regulations.

Patient Confidentiality: prior to publication, patient data listings will be removed to ensure that patient confidentiality is maintained.

In alignment with the EU Directive 95/46/EC and Israeli MOH Regulations on data protection, the patient's name will not be identifiable from the subject study code. The investigator should keep the code given in the "Patient Identification Log" (see Section 16.4.5).

After obtaining patient's consent, the investigator will permit the study monitor, independent auditor, or regulatory agency personnel to review the portion of the patient's medical record that is directly related to the study. This shall include all study relevant documentation including patient medical history to verify eligibility, laboratory tests results, admission/discharge summaries for hospital admissions occurring while the patient is enrolled in the study, and autopsy reports for deaths occurring during the study (if applicable).

14.6 Liability And Insurance

A certificate of clinical trials insurance will be provided according to the Israeli Ministry of Health regulations to the study center by ENOX.

15. DOCUMENTATION

15.1 Study File and Site Documents

Prior to the initiation of the study, the following items must be received by the sponsor from the site:

1) Confidential disclosure agreement.
2) Signed protocol, amendment(s) and notification(s) page(s).
3) The principal investigator's curriculum vitae.
4) Completed and signed required regulatory form (a complete submission package).
5) Signed clinical study agreement.
6) IRB-written approval for the protocol, amendment(s), informed consent form, patient Information Sheet.
7) EC/IRB Membership list or an official statement from the EC/IRB stating the EC/IRB is in compliance with ICH- GCP guidelines.
8) Financial disclosure information for all persons listed in study delegation form.
9) Bacteriologies laboratory quality documentation.
10) Relevant normal ranges of all laboratory tests done to study participants.

15.2 Study Documents Supplied by the Sponsor

The sponsor will supply the investigator with the following items:

- Current version of the Investigator's Brochure
- Printed CRF and the electronic CRF in PDF format (Master CRF)
- Informed consent form – using the Israeli Ministry of Health template
- Insurance certificate
- Operations manual - for all study specific equipment

15.3 Maintenance and Retention of Records

It is the responsibility of the Investigator to maintain a comprehensive and centralized filing system of all relevant documentation.

- Investigators will be instructed to retain all study records required by the sponsor and regulatory authorities in a secure and safe facility with limited access for one of the following time periods based on notification from the Sponsor
- A period of at least two years from last marketing authorization and notification from the sponsor
- Or a period of at least 15 years after discontinuation of clinical development of the investigational product as confirmed by the Sponsor
- Or longer if required by local regulations

The investigator will be instructed to consult with the sponsor before disposal of any study records and to provide written notification to the sponsor of any change in the location, disposition, or custody of the study files.

16. DATA HANDLING

16.1 Data Collection via Paper CRF

CRFs for individual patients will be provided by the sponsor. The CRFs are supplied on NCR paper; one copy will be kept by the investigator and the original and second copy will be retrieved by the ENOX study monitor.

CRFs are used to record study data and are an integral part of the study and subsequent reports. The data requested on the CRF must be recorded in black/blue ink (hard point pen) as the study is in progress. Additional information may be included as warranted. Any correction or deletion should be made by drawing a single line through the entry so that the original entry is still legible; this change must be initialed and dated by study staff personnel. Changes regarding AEs and clinical judgments must be initialed and dated by the investigator.

CRFs will be considered complete when all missing and/or incorrect data have been accounted for. Copies of the study records must be retained in the files of the investigator in accordance local regulations.

Completed CRFs must be signed by the investigator for each patient enrolled, including those prematurely terminated from the study for any reason. The reason for premature termination must be recorded by the investigator for each patient, on the termination visit CRF page.

CRFs must be kept current to reflect the patient's status at each phase during the course of the study. Patients should not be identified by name; appropriately coded identification and patient's non-identifiable initials must be used. The Investigator must keep a separate log of the patients' names, used initials and addresses and it must be maintained confidential.

CRFs must be completed and available for on-site review at each monitoring visit and within 14 working days of a patient's termination from the study.

All source documents, such as clinic charts, must be available to allow source data verification.

CRFs will be sent to the DMC (Data Management Center- Medistat) promptly after the visit has been monitored.

16.2 Data Entry

Data entry system reflecting the CRFs will be prepared prior to study data entry stage.

Independent persons will enter data into the database system using double data entry techniques. Data identified as erroneous, or key data that are missing, will be referred to the CRA or, if necessary, to the Investigator for resolution on data query forms. On return of these forms, the database will be amended. Prior to the closure of the database, a quality control check of the raw data will be performed. All errors detected during the audit will be corrected prior to database closure.

16.3 Medical Information Coding

Coding of AEs will be performed automatically by Medistat, using the Medical Dictionary for Drug Regulatory Activities (MedDRA) dictionary, using the most updated version. PT (preferred term) and SOC (System Organ Class) will be used.

The AE as well as SAE codes will be approved by the Sponsor.

Similarly, coding of all medications will occur using the WHO (World Health Organization) Drug dictionary. SAEs will be coded using MedDRA.

16.4 Data Validation

Visual and computerized methods of data validation will be applied in order to ensure accurate, consistent, and reliable data for the subsequent statistical analysis. These procedures aim to detect out-of-range values, contradictory data, and abnormal evolutions over time, and possible undetected protocol violations (eligibility criteria, time and medication compliance, etc.).

16.4.1 Data Correction

After the data have been entered and verified, various edit checks will be performed for the purpose of ensuring the accuracy, integrity, and validity of the database. These edit checks may include:

- Missing value checks
- Range checks
- Consistency checks
- Sequence checks
- Probabilistic checks
- Protocol adherence checks

16.4.2 Data Queries

Data identified as erroneous, or key data that are missing, will be referred to the CRA or, if necessary, to the Investigator for resolution on data query forms for prompt resolution.

Queries will be generated after the second data entry is completed. Queries in a PDF format will be emailed to the sites. The emails will copy the CRA.

The resolved queries with the investigator's signature will be faxed to MediStat. The original documents will be mailed later.

On return of these forms, the database will be amended. All data modifications resulting from review or querying of the data will be electronically tracked.

Any errors detected by either the study monitor or the Investigator after query, resolution should be documented in CRF data change forms.

All the queries will be attached to the CRFs.

In all cases the signature of an investigator or designee and of the study Monitor will be required.

Prior to the closure of the database, a quality control check of the raw data will be performed. All errors detected during the audit will be corrected prior to database closure

16.4.3 Data Extract

All database tables are kept as raw data in SAS format. Each table (file) contains all the visits of the corresponding form.

16.4.4 Source Documents

Prescription forms, label logs and laboratory test results, and all other source documents should be maintained and kept at the study site in the patient study binder.

16.4.5 Additional Documents and Records

1) Patient screening log and patient assignment log – A listing of all patients who were screened, and those who have signed the informed consent form, respectively.

2) Patient identification log - This allows linking of the enrolled patient medical records to the study documents. Information should include, but is not limited to: patient's ID number, patient's name, date of birth, contact information, patient's non-identifiable study code. This list will be maintained by the investigational site.

17. QUALITY ASSURANCE AUDITS

17.1 Good Clinical Practice

The study described in this protocol will be carried out according to the local regulatory requirements (Israeli ministry of health) and FDA, ICH accepted standards of Good Clinical Practice. All procedures described in this protocol will be performed according to approved written Standard Operating Procedures unless otherwise stated.

17.2 Quality Laboratory Standards

Bacteriologies laboratory tests/evaluations described in this protocol will be conducted in the hospital laboratories, accordance with quality laboratory standards as requested by the laboratory quality plan.

17.3 Quality Assurance Program

There is no sponsor-audit planned in this study.

17.4 Regulatory Inspections

The study may be inspected by regulatory agencies. These inspections may take place at any time during or after the study and are based on national regulations, as well as ICH guidelines.

18. STUDY MONITORING

18.1 Monitors/CRAs and Monitoring Visits

The study monitor/CRA will be responsible for ensuring adherence to Israeli Ministry of Health Regulations, ICH guidelines and the ENOX Standard operating procedures. Study Monitors for this trial will be provided by the Sponsor. The monitors will follow the current "Guideline for the Monitoring of Clinical Investigatsor" supplied by the FDA or will operate according to the EU Directives and in compliance with ICH guidelines.

Experienced independent monitors or monitors from CROs will also be trained in Physio-Logic Ltd SOPs, study protocol and the study monitoring conventions.

Regular monitoring of study data at the site will be performed as defined by the study specific monitoring plan. The site will be monitored to verify that enrolment rate, data recording, and protocol adherence are satisfactory. The frequency of monitoring may fluctuate depending upon enrolment rate, quantity of data collected and the complexity of the study, and will be described in the monitoring plan.

These monitoring visits will be performed for the purpose of verifying adherence to the protocol and the completeness and accuracy of data entered on CRFs. The study monitor will verify CRF entries by comparing them with the primary source documents (hospital/clinic/office records), which will be made available for this purpose. The monitor will review the maintenance of regulatory documentation. The Monitor will review the progress of the study with the investigator and other site personnel on a regular basis. Case report form sections may be collected during these visits. At the end of the study, a close-out monitoring visit will be performed. Monitoring visits will be arranged in advance with site personnel at a mutually acceptable time. Sufficient time must be allowed by the site personnel for the monitor to review CRFs and relevant source documents. The coordinator and/or investigator should be available to answer questions and provide clarifications, as needed.

18.2 Primary Source Documents

The investigator must maintain primary source documents to support CRF data entries. These documents, which are considered "source data", may include but are not limited to:

- Demographic information
- Evidence supporting the diagnosis/condition for which the patient is being studied
- General information supporting the patient's participation in the study

- Medical history and physical findings
- Hospitalization or emergency room records (if applicable)
- Each study visit by date, including dates of catheter placement and removal, any relevant findings/notes by the investigator(s), and occurrence (or lack) of adverse events.
- Any additional visits during the study
- Any relevant telephone conversations with the patient regarding the study or possible adverse events
- Original, signed informed consent forms for study participation

The investigator must also retain all patient specific printouts/reports of tests/ procedures performed as a requirement of the study. During monitoring visits the monitor will need to verify data in the CRFs against these source data.

19. USE OF INFORMATION AND PUBLICATION

Confidential Information

All information supplied by ENOX in connection with this study and not previously published, is considered confidential information. This information includes, but is not limited to, the investigators' brochure, clinical protocol, case report forms and other scientific data. Any data collected during the study are also considered confidential. This confidential information shall remain the sole property of ENOX, shall not be disclosed to others without a written consent of ENOX, and shall not be used except in the performance of this study.

The information developed during the conduct of this clinical study is also considered confidential, and will be used by ENOX in connection with the development of the medical device. The information may be disclosed as deemed necessary by ENOX. To allow the use of the information derived from this clinical study, the investigator is obliged to provide ENOX with complete test results and all data developed in this study. The information obtained during this study may be made available to other investigators who are conducting similar studies.

Should the investigator wish to publish the results of this study, the investigator agrees to provide ENOX with a manuscript for review 60 (sixty) days prior to submission for publication.

ENOX retains the right to delete from the manuscript information that is confidential and proprietary and to object to suggested publication and/or its timing (at the company’s sole discretion).

In the event that ENOX chooses to publish the data from this study, a copy will be provided to the investigator at least 30 days prior to the expected date of submission to the intended publisher.

20. STUDY PERSONNEL

20.1 Investigative Site

The Principal Investigator

The principal investigator will have overall responsibility to lead the site study team and all aspects of the study in Beilinson. The Principal Investigator will oversee the accrual of appropriate patients, the conduct of the study according to the trial protocol, and the collection of required data.

Study Coordinator

A study coordinator or other staff member may be designated by the principal investigator to be responsible for patient scheduling and completion of all patients' case report forms and recording of adverse events. He/she will forward blood samples and requests to the appropriate laboratories, will obtain and forward laboratory results, and perform other duties delegated by the principal investigator as instructed.

20.2 Data Management and BioStatistics

Medistat is responsible for adequate performance of the data management application using SAS; the conduct of the routine data management procedures; and the performance of the statistical analysis as defined in this protocol.

20.3 Monitor/Clinical Research Associate

The Monitor/Clinical Research Associate (CRA) is responsible for monitoring the conduct of the study at the study centers. Monitoring visits will be arranged in advance, at a mutually acceptable time, with site personnel.

Monitoring is done by Enox personnel

The Medical Monitor is responsible for periodically reviewing all safety clinical data.

21. REFERENCE LIST

1 Hall, C.B.*, et al.* (2009) The burden of respiratory syncytial virus infection in young children. *The New England journal of medicine* 360, 588-598
2 Klevens, R.M.*, et al.* (2007) Estimating health care-associated infections and deaths in U.S. hospitals, 2002. *Public Health Rep* 122, 160-166
3 Stockman, L.J.*, et al.* (2012) Respiratory syncytial virus-associated hospitalizations among infants and young children in the United States, 1997-2006. *The Pediatric infectious disease journal* 31, 5-9
4 Hooton, T.M.*, et al.* (2010) Diagnosis, prevention, and treatment of catheter-associated urinary tract infection in adults: 2009 International Clinical Practice Guidelines from the Infectious Diseases Society of America. *Clin Infect Dis* 50, 625-663
5 Bi, X.C.*, et al.* (2009) Pathogen incidence and antibiotic resistance patterns of catheter-associated urinary tract infection in children. *Journal of chemotherapy* 21, 661-665
6 Ronald, A. (2002) The etiology of urinary tract infection: traditional and emerging pathogens. *The American journal of medicine* 113 Suppl 1A, 14S-19S
7 Costerton, J.W.*, et al.* (1999) Bacterial biofilms: a common cause of persistent infections. *Science* 284, 1318-1322
8 Dunne, W.M., Jr. (2002) Bacterial adhesion: seen any good biofilms lately? *Clinical microbiology reviews* 15, 155-166
9 Webb, J.S.*, et al.* (2003) Bacterial biofilms: prokaryotic adventures in multicellularity. *Curr Opin Microbiol* 6, 578-585
10 Ha, U.S. and Cho, Y.H. (2006) Catheter-associated urinary tract infections: new aspects of novel urinary catheters. *Int J Antimicrob Agents* 28, 485-490
11 Johnson, J.R.*, et al.* (2006) Systematic review: antimicrobial urinary catheters to prevent catheter-associated urinary tract infection in hospitalized patients. *Ann Intern Med* 144, 116-126
12 Ramritu, P.*, et al.* (2008) A systematic review comparing the relative effectiveness of antimicrobial-coated catheters in intensive care units. *Am J Infect Control* 36, 104-117
13 Siddiq, D.M. and Darouiche, R.O. (2012) New strategies to prevent catheter-associated urinary tract infections. *Nature reviews. Urology* 9, 305-314
14 Regev-Shoshani, G.*, et al.* (2010) Slow release of nitric oxide from charged catheters and its effect on biofilm formation by Escherichia coli. *Antimicrobial agents and chemotherapy* 54, 273-279
15 Carlsson, S.*, et al.* (2005) Intravesical nitric oxide delivery for prevention of catheter-associated urinary tract infections. *Antimicrob Agents Chemother* 49, 2352-2355
16 Regev-Shoshani, G.*, et al.* (2011) Comparative efficacy of commercially available and emerging antimicrobial urinary catheters against bacteriuria caused by E. coli in vitro. *Urology* 78, 334-339
17 AW, S. and AJ, V. (1977) Solubility of nitric oxide in aqueous and nonaqueous solvents. *J Chem Soc Faraday Trans*, 18:1239-1244
18 Moncada, S.*, et al.* (1991) Nitric oxide: physiology, pathophysiology, and pharmacology. *Pharmacological reviews* 43, 109-142
19 De Groote, M.A. and Fang, F.C. (1995) NO inhibitions: antimicrobial properties of nitric oxide. *Clinical infectious diseases : an official publication of the Infectious Diseases Society of America* 21 Suppl 2, S162-165
20 Witte, M.B. and Barbul, A. (2002) Role of nitric oxide in wound repair. *American journal of surgery* 183, 406-412
21 Fang, F.C. (1997) Perspectives series: host/pathogen interactions. Mechanisms of nitric oxide-related antimicrobial activity. *The Journal of clinical investigation* 99, 2818-2825

22 Hibbs, J.B., Jr*., et al.* (1992) Evidence for cytokine-inducible nitric oxide synthesis from L-arginine in patients receiving interleukin-2 therapy. *The Journal of clinical investigation* 89, 867-877
23 Schairer, D.O*., et al.* (2012) The potential of nitric oxide releasing therapies as antimicrobial agents. *Virulence* 3, 271-279
24 Wink, D.A*., et al.* (1991) DNA deaminating ability and genotoxicity of nitric oxide and its progenitors. *Science* 254, 1001-1003
25 Laval, F*., et al.* (1997) A discussion of mechanisms of NO genotoxicity: implication of inhibition of DNA repair proteins. *Rev Physiol Biochem Pharmacol* 131, 175-191
26 Rubbo, H*., et al.* (1994) Nitric oxide regulation of superoxide and peroxynitrite-dependent lipid peroxidation. Formation of novel nitrogen-containing oxidized lipid derivatives. *J Biol Chem* 269, 26066-26075
27 Wink, D.A. and Mitchell, J.B. (1998) Chemical biology of nitric oxide: Insights into regulatory, cytotoxic, and cytoprotective mechanisms of nitric oxide. *Free Radic Biol Med* 25, 434-456
28 Morris, S.L. and Hansen, J.N. (1981) Inhibition of Bacillus cereus spore outgrowth by covalent modification of a sulfhydryl group by nitrosothiol and iodoacetate. *J Bacteriol* 148, 465-471
29 Barraud, N*., et al.* (2009) Nitric oxide-mediated dispersal in single-and multi-species biofilms of clinically and industrially relevant microorganisms. *Microbial biotechnology* 2, 370-378
30 Hakim, T.S*., et al.* (1996) Half-life of nitric oxide in aqueous solutions with and without haemoglobin. *Physiological measurement* 17, 267-277
31 Hughes, M.N. Chemistry of Nitric Oxide and
Related Species. *Methods in Enzymology* 436, 3-16
32 Franken, A. and Bosch, E.E.M.v.d. (2007) Anti-microbial coatings for urological applications. *European Cells and Materials* 14, 130
33 Desai, D.G*., et al.* (2010) Silver or nitrofurazone impregnation of urinary catheters has a minimal effect on uropathogen adherence. *The Journal of urology* 184, 2565-2571
34 Donlan, R.M. and Costerton, J.W. (2002) Biofilms: survival mechanisms of clinically relevant microorganisms. *Clinical microbiology reviews* 15, 167-193
35 Charville, G.W*., et al.* (2008) Reduced bacterial adhesion to fibrinogen-coated substrates via nitric oxide release. *Biomaterials* 29, 4039-4044
36 Darling, K.E. and Evans, T.J. (2003) Effects of nitric oxide on Pseudomonas aeruginosa infection of epithelial cells from a human respiratory cell line derived from a patient with cystic fibrosis. *Infection and immunity* 71, 2341-2349
37 Nablo, B.J*., et al.* (2001) Sol-gel derived nitric-oxide releasing materials that reduce bacterial adhesion. *J Am Chem Soc* 123, 9712-9713
38 Hermann, M*., et al.* (2006) Nitric oxide in hypertension. *Journal of clinical hypertension* 8, 17-29
39 Gries, A*., et al.* (1998) Inhaled nitric oxide inhibits human platelet aggregation, P-selectin expression, and fibrinogen binding in vitro and in vivo. *Circulation* 97, 1481-1487
40 Kermarrec, N*., et al.* (1998) Impact of inhaled nitric oxide on platelet aggregation and fibrinolysis in rats with endotoxic lung injury. Role of cyclic guanosine 5'-monophosphate. *American journal of respiratory and critical care medicine* 158, 833-839
41 Paoloni, J.A*., et al.* (2003) Topical nitric oxide application in the treatment of chronic extensor tendinosis at the elbow: a randomized, double-blinded, placebo-controlled clinical trial. *The American journal of sports medicine* 31, 915-920
42 Aspevall, O*., et al.* (2001) European guidelines for urinalysis: a collaborative document produced by European clinical microbiologists and clinical chemists under ECLM in collaboration with ESCMID. *Clinical microbiology and infection : the official publication of the European Society of Clinical Microbiology and Infectious Diseases* 7, 173-178

22. APPENDICES

Appendix 1

Microscopic Examinations:

Upon removal, the indwelling urinary catheter will be cut into 1-cm sections and the balloon section. Samples for EM will be cut into two transverse sections. One section of the urinary catheter is collected into an individual 50ml vial. Each vial is labeled by study coordinator: No., Group No., patient No., date of removal & collection time.

Immediately following collection, all the vials are stored at 2-8°C until their transportation to microbiology lab for bacteriological analysis.

The other section of the indwelling urinary catheter is collected into an individual 50ml vial, containing fixative (supplied by sponsor) for EM.

Each vial is labeled by study coordinator: Group No., patient No., date of removal & collection time.

Immediately following collection, all the vials are stored at 2-8°C and transported to the microbiology laboratory. In case transportation is delayed the vials can be stored overnight at 2-8°C and transported the next day for microbial and biofilm analysis.

Appendix 2

Ministry of Health
Health Technology and Infrastructure Administration
Medical Devices Department

מדינת ישראל
STATE OF ISRAEL

משרד הבריאות
המנהל לטכנולוגיות רפואיות ותשתיות
היחידה לאביזרים ומכשירים רפואיים

אישור רישום בפנקס האביזרים והמכשירים הרפואיים

ניתן בזאת אישור , כי בהתאם לבקשת רישום מס : 880000
האביזרים / מכשירים רפואיים (אמ"ר) הבאים :

שם האמ"ר	ציוד רפואי לשימוש חד פעמי - ראה רשימה מצורפת Disposable medical devices - see attached list
יעוד האמ"ר	ראה רשימה מצורפת
התויה	1. בי"ח - כללי -
שם בעל רישום וכתובתו	ביומטריקס בע"מ ; קרית מדע 4, ירושלים 97776 ; ישראל
שם יצרן וכתובתו	ביומטריקס בע"מ ; קרית המדע 4, הר חוצבים ; ישראל
שם אתר יצור וכתובתו	ביומטריקס בע"מ ; קרית המדע 4, הר חוצבים ; ישראל

התניות

הנחיות

- לפי הוראות היצרן שאושרו ע"י גוף המאשר: Intertek 0473
- אישור בהתאם לאישור CE ומערכת איכות בתוקף
- הערות נוספות: מצורפים לאישור זה 2 דפים נספחים של מוצרים מאושרים.
- מאושר לשימוש בהתאם להוראות היצרן כפי שאושרו על ידי הגוף המאשר הנ"ל, לרופא(ה) או אח(ות) בבית חולים בלבד

נרשמו בפנקס האביזרים והמכשירים הרפואיים במשרד הבריאות
תוקף האישור לשיווק האמ"ר הינו ליעודים ולהתוויות המתוארים לעיל בלבד
האישור בתוקף עד :31/07/2015

ד"ר שרית סיון
יחידת האמ"ר

27/08/2013

שם ותפקיד המאשר | תאריך חתימת האישור | חתימה

BIOMETRIX
CRITICAL CARE SOLUTIONS

Biometrix ltd | Kiryat Mada 4, P.O.Box 45057, Jerusalem 91450, Israel
Tel: 972-2-5861241 | Fax: 972-2-5861322
| www.biometrixmedical.com mail@biometrixmedical.com

רשימת מוצרים	CE Certificate #
**1. Chest drainage systems:**	
· Reservoirs	242 CE
· Drains & catheters: thoracic, trocar, pneumothorax	242 CE
**2. Drainage systems:**	
· Reservoirs: URX, Bulbs, minivac, prevacuum	242 CE
· Tissue drains (round, flat, fluted including trocars)	242 CE
· Suction tubing and probes	242 C CE
**3. Physiological/blood pressure monitoring systems and accessories (including cables):**	
· Arterial line	242 CE
· ICP	242 CE
· IUP	242 CE
· Intra-abdominal pressure	242 CE
**4. Cardioplegia: tubing systems and connectors for cardiopulmonary bypass.**	242 CE
**5. Angio sets & accessories:**	
· PTCA	242 CE
· Cath lab & angio packs	242 CE
· Guide wires	242 CE
· Manifold sets (systems for contrast media injection including high pressure lines)	242 CE
· Angiographic syringes	242 CE
**6. Infusion and irrigation lines** (including tubing sets, stopcocks, connectors, luer activated and infusion needles)	242 CE
**7. Vascular catheters:**	
· CVC	242 CE
· Dialysis	242 CE
· Arterial	242 CE
· Introducers	242 CE
· High flow	242 CE

נספח מס' 1 מתוך 2

לאישור רישום בפנקס האמ"ר

מס. 880000

מדינת ישראל
משרד הבריאות
MEDICAL DE...
MINISTRY
STATE OF ISRAEL

BIOMETRIX
CRITICAL CARE SOLUTIONS

Biometrix ltd | Kiryat Mada 4, P.O.Box 45057, Jerusalem 91450, Israel
Tel: 972-2-5861241 | Fax: 972-2-5861322
| www.biometrixmedical.com mail@biometrixmedical.com

**8. Surgical Procedure Packs:**	
· Drapes, gowns & covers	**242 CE**
· Electrosurgical pencil	**242 CE**
· Surgical instruments (scalpels, forceps etc.)	**242 CE**
· Identiloops & silclamps	**242 CE**
· Accessories/ widgets\	**242 CE**
· Ophthalmic surgery instruments	**242 CE**
**9. Electronic measuring devices: FSPM**	**242 B CE**
10. **Drainage catheters**: foley, nephrostomy, biliary and more (suprapubic etc.)	**242 CE**

נספח מס׳......2......מתוך.......2......
לאישור רישום בפנקס האמ״ר
מס. 880600

מדינת ישראל
משרד הבריאות
אמ״ר
אביזרים ומכשירים רפואיים
MEDICAL DEVICES
MINISTRY OF HEALTH
STATE OF ISRAEL

Appendix 3

No-charged catheter

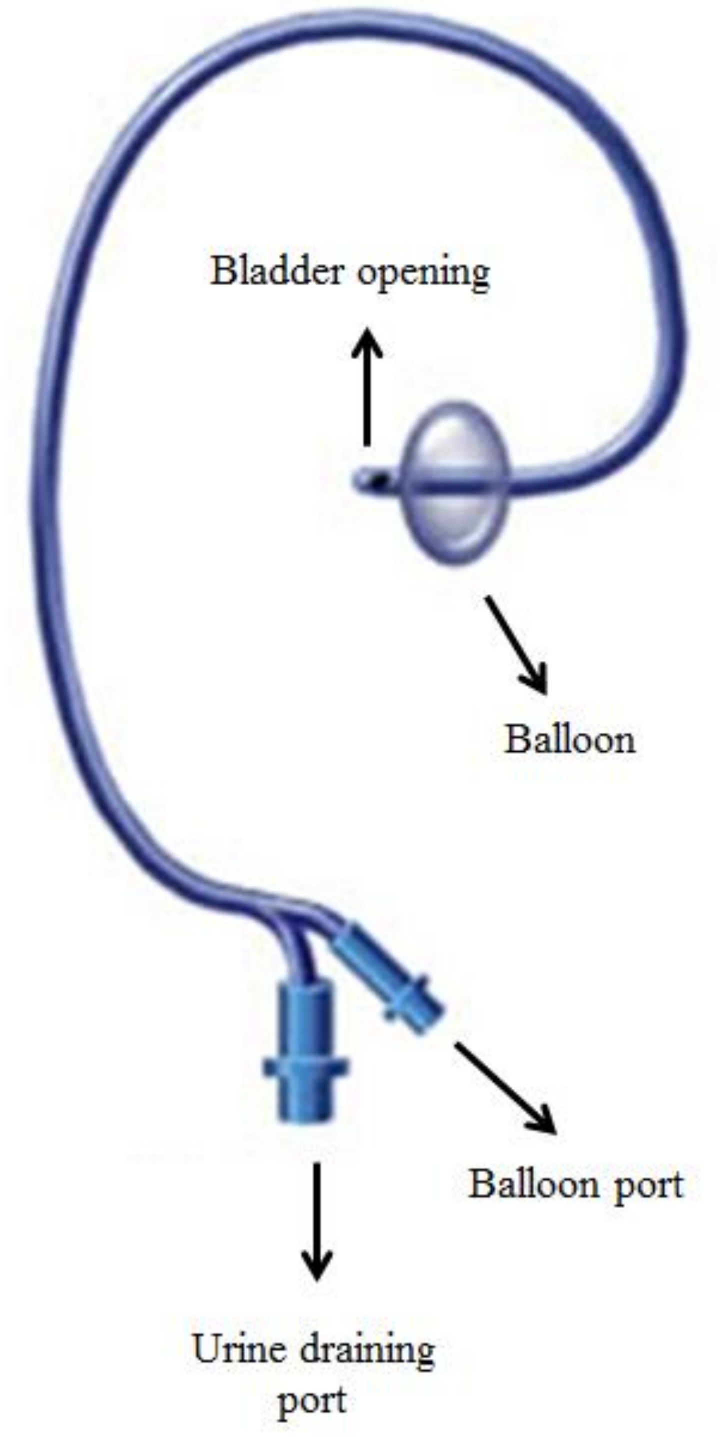

Appendix 4

Date of Receiving: July 01, 2014

Date of Testing: July 16, 2014 Test No.: 46344

Company: Name: ENOX Ltd Code: 1275

Address: Kibbutz Bet Alfa
19140 Israel

Contact: Dan Saden

Sample: Enox Impregnated Catheter

Batch No.: 004

Test Report Version No.: 1

Hy Laboratories Ltd.
Park Tamar, Rehovot, 7670606 Israel
Tel. 972-8-9366475
Fax. 972-8-9366474
hy·labs®

STERILITY TEST FORM

THE STANDARDS INSTITUTION OF ISRAEL

ISRAC Israel Laboratory Accreditation Authority ISO/IEC 17025 No. 184

Method: Hy Laboratories SOP No. 10-004

Technique: Direct Transfer or Immersion Technique (Only TSB).

Date Started : July 16, 2014 **Date of Completion :** July 30, 2014

Item	Lot #	No. of Samples	Sterility Test Results			
			Intermediate Results Date Time /		Final Results Date: 30.07.14 Time: 11:00	
			Growth	No Growth	Growth	No Growth
Enox Impregnated Catheter	004	1	☐	☐	☐	☒
Enox Impregnated Catheter	004	1	☐	☐	☐	☒
Enox Impregnated Catheter	004	1	☐	☐	☐	☒
Enox Impregnated Catheter	004	1	☐	☐	☐	☒
Enox Impregnated Catheter	004	1	☐	☐	☐	☒
Enox Impregnated Catheter	004	1	☐	☐	☐	☒
Enox Impregnated Catheter	004	1	☐	☐	☐	☒
Enox Impregnated Catheter	004	1	☐	☐	☐	☒
Enox Impregnated Catheter	004	1	☐	☐	☐	☒
Enox Impregnated Catheter	004	1	☐	☐	☐	☒

Intermediate Results:

Technician: ____/____ Reviewed by: ____/____ Date: ____/____

Test Conclusion:

The Product Complies With the Test: ☒ Yes / ☐ No

GPT of Medium for performing sterility test:

Medium	Organism	Strain		
Soybean - Caseine Digest Batch # : 230070	*Bacillus subtilis*	ATCC 6633	+	+
	Candida albicans	ATCC 10231	+	+
	Aspergillus brasiliensis	ATCC 16404	+	+
	Staphylococcus aureus	ATCC 6538	+	+
	Pseudomonas aeruginosa	ATCC 9027	+	+

Negative control of culture Media: TSB: ☐ Growth / ☒ No Growth

Technician: Katya Zaharov Microbiology Technician **Date:** July 30, 2014

Approved by: GALINA KOGAN Supervisor **Date:** July 30, 2014

This report is submitted for the exclusive use of the person or corporation to whom is addressed, and neither the report nor the name of these laboratories nor any members of its staff may be used in connection with the advertising, sale or any product or process without the written permission of Hy-Laboratories Ltd. The results are approved for samples tested only.

The use of ISRAC symbol relates to tests/calibrations which are included in organization scope of accreditation and performance according to the accreditation rules of the tests performance.

ISRAC is not responsible for the results of the tests performed by the organization/research facility and accreditation/recognition does not constitute a certificate approval of any item, system or process tested

This certificate need to related in full and no part thereof shell be quoted in other documents.

Appendix 5

Possible adverse events related to NO-charged catheters

No.	Adverse Event	Risk analysis
1	Decrease in systemic blood pressure	In light of the low concentration expected to be released from the catheter (up to 5 ppm NO), and in light of the short half-life of NO in the blood, we believe that such concentration (4-16 fold lower than the concentration given to neonates with pulmonary hypertension) is unlikely to decrease systemic blood pressure. Blood pressure will be assessed 3 times per day during hospitalization (every 8 hours), on catheter removal day, on every unscheduled visit, and 30 days after catheterization.
2	Increased bleeding time	Blood coagulation tests (Prothrombin Time, Activated Partial Thromboplastin Time, and International Normalized Ratio) will be performed on a routine basis during hospitalization (every day), on catheter removal day, on every un-scheduled visit, and 30 days after catheterization.
3	Erection	A physical examination with emphasis on the genitourinary system (penis, glans, shaft, and prepuce) will be performed on a daily basis during hospitalization, on catheter removal day, on every un-scheduled visit, and 30 days after catheterization.
4	Local irritation	Biocompatibility of NO-charged catheters was evaluated by several tests conducted in animals: in vivo intradermal reactivity tests, in vivo sensitization test and in vivo safety trial for NO-charged catheters (see appendix 9, 10 and 2, in investigators brochure, respectively). All tests revealed that NO-charged catheter does not trigger dermal reaction in terms of erythema and edema. However, since NO-charged catheters will be used for the first time in human during this trial we have to consider the possibility of local irritation.<br>A physical examination with emphasis on the genitourinary system (penis, glans, shaft, and prepuce) will be performed on a daily basis during hospitalization, on catheter removal day, on every un-scheduled visit, and 30 days after catheterization.

Severe adverse events which are life threatening, and/or can result in prolongation of patient hospitalization, and/or persistent or significant disability, and/or death are not expected in this study.
